# Site-selective protein conjugation at histidine†
†Electronic supplementary information (ESI) available. See DOI: 10.1039/c8sc03355b


**DOI:** 10.1039/c8sc03355b

**Published:** 2018-10-09

**Authors:** Karolina Peciak, Emmanuelle Laurine, Rita Tommasi, Ji-won Choi, Steve Brocchini

**Affiliations:** a UCL School of Pharmacy , University College London , 29-39 Brunswick Square , London , WC1N 1AX , UK . Email: steve.brocchini@ucl.ac.uk; b Abzena , Babraham Research Campus, Babraham , Cambridge CB22 3AT , UK

## Abstract

Site-selective conjugation generally requires both (i) molecular engineering of the protein of interest to introduce a conjugation site at a defined location and (ii) a site-specific conjugation technology. Three N-terminal interferon α2-a (IFN) variants with truncated histidine tags were prepared and conjugation was examined using a bis-alkylation reagent, PEG_(10kDa)_-mono-sulfone **3**. A histidine tag comprised of two histidines separated by a glycine (His_2_-tag) underwent PEGylation. Two more IFN variants were then prepared with the His_2_-tag engineered at different locations in IFN. Another IFN variant was prepared with the His-tag introduced in an α-helix, and required three contiguous histidines to ensure that two histidine residues in the correct conformation would be available for conjugation. Since histidine is a natural amino acid, routine methods of site-directed mutagenesis were used to generate the IFN variants from *E. coli* in soluble form at titres comparable to native IFN. PEGylation conversions ranged from 28–39%. A single step purification process gave essentially the pure PEG–IFN variant (>97% by RP-HPLC) in high recovery with isolated yields ranging from 21–33%. The level of retained bioactivity was strongly dependent on the site of PEG conjugation. The highest biological activity of 74% was retained for the PEG_10_-106(**H**G**H**G)-IFN variant which is unprecedented for a PEGylated IFN. The His_2_-tag at 106(**H**G**H**G)-IFN is engineered at the flexible loop most distant from IFN interaction with its dimeric receptor. The biological activity for the PEG_10_-5(**H**G**H**)-IFN variant was determined to be 17% which is comparable to other PEGylated IFN conjugates achieved at or near the N-terminus that have been previously described. The lowest retained activity (10%) was reported for PEG_10_-120(**HHH**)-IFN which was prepared as a negative control targeting a IFN site thought to be involved in receptor binding. The presence of two histidines as a His_2_-tag to generate a site-selective target for bis-alkylating PEGylation is a feasible approach for achieving site-selective PEGylation. The use of a His_2_-tag to strategically engineer a conjugation site in a protein location can result in maximising the retention of the biological activity following protein modification.

## Abstract 2

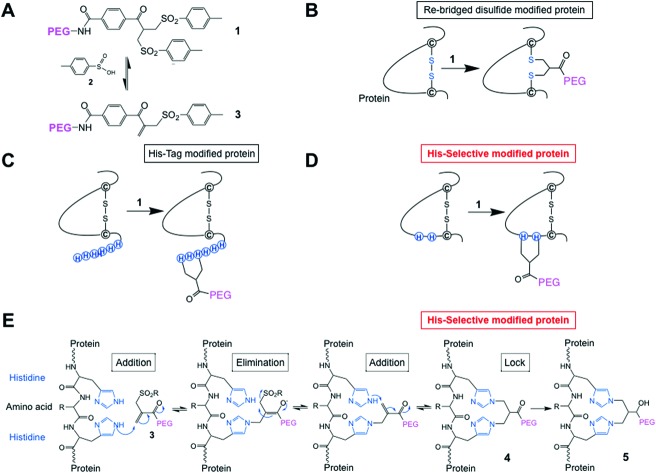
Site-selective conjugation generally requires both (i) molecular engineering of the protein of interest to introduce a conjugation site at a defined location and (ii) a site-specific conjugation technology.

## Introduction

The functionality and efficacy of therapeutic proteins can be increased by the covalent conjugation of drugs, probes and polymers (*e.g.* antibody drug conjugates (ADCs) and PEGylation). Most proteins will have regions and sites in their structure where conjugation can result in optimal stability, activity, and pharmacokinetics.^1^–^3^ Computational strategies are being used to identify sites in the protein structure to predict the impact of PEGylation.^1^ Site-selective conjugation generally requires both (i) molecular engineering of the protein of interest to introduce a conjugation site at a defined location and (ii) a site-specific conjugation technology.^4^

Many conjugation methods have been described^5^,^6^ and some^7^–^13^ can be specific for an amino acid residue or a glycosyl moiety. Cysteine incorporation into a protein as a site for conjugation has been described in many preclinical studies^14^,^15^ including studies where large numbers of variants have been designed to determine the best site for an unpaired cysteine.^16^ Introduction of an additional cysteine can cause scrambling of the native disulfides, and protein dimerisation, oxidation, and aggregation.^17^,^18^ The presence of an accessible cysteine for conjugation can also cause protein aggregation during downstream processing by forming intermolecular disulfides. The unpaired single cysteines engineered into a protein can also be blocked for conjugation by cysteinylation and glutathionylation, which are referred to as ‘cysteine capping’.^19^ These oxidised forms of the added cysteine must be reduced before conjugation can be conducted, which can often be difficult to accomplish without reducing natural disulfide bonds that may be present in the protein. Incorporation of an unpaired cysteine for conjugation is best utilised in proteins that do not have a native disulfide.

Site-selective conjugation can be achieved by the incorporation of noncanonical amino acids (ncAA), which are sometimes referred to as non-natural amino acids (NNAAs).^20^–^25^ As with cysteine incorporation, ncAA incorporation can be achieved by addition or substitution of a single amino acid into the protein of interest. ncAA incorporation is typically achieved by genetically reassigning a stop codon as a recognition site for an engineered pair of tRNA and aminoacyl-tRNA synthetase that incorporates the ncAA at the stop codon.^26^ However, protein expression using stop codon reassignment can result in “leak expression”^27^ and low yields of protein expression^28^ due to low overall suppression efficiency defined as the percentage of stop codon read-through, and due to mechanisms that degrade mRNA^29^ containing premature stop codons, called nonsense mediated mRNA decay. Strategies have been described^1^,^26^,^30^ such as using cell-free protein synthesis to address limitations^31^ for the specific incorporation of ncAAs.

ncAAs have side chains (*e.g.* keto, azide or alkyne) with orthogonal reactivity to native amino acids.^20^–^25^ Conjugation conversions and yields with ncAA modified proteins can be high^32^ depending on many factors including reagent stoichiometry and the presence of catalysts (*e.g.* copper). Orthogonal reaction conditions must be suitable for protein modification. For example, the use of copper in click approaches can be challenging due to toxicity^33^ and can cause protein misfolding, aggregation and degradation.^34^ Copper readily complexes to proteins and is the basis for quantification assays, so it is not clear why there has been so much effort to use copper in conjugation reactions with proteins.

Strain-promoted azide alkyne cycloadditions (SPACC) is one alternative to avoiding copper. However, hydrophobicity caused by conjugation linker moieties^35^ or due to surface accessible ncAAs can cause non-specific protein binding.^36^ Although the impact of hydrophobicity will be less at the site of conjugation for PEGylation applications, ADC hydrophobicity has also long been recognised to cause accelerated clearance and reduced efficacy.^37^,^38^ ADC hydrophobicity is also caused by linker–drug structure and the drug–antibody ratio (*i.e.* drug loading). Strategies have been described to solve the challenges caused by hydrophobicity to (i) reduce non-specific binding in azide alkyne cycloadditions^39^,^40^ and (ii) optimise ADC development.^41^,^42^

Increased conjugation selectivity can also be accomplished by removal of amino acid residues that undergo competitive conjugation. This strategy was followed for the development of PEGvisomant,^43^ which is a growth hormone antagonist. Lysine residues were substituted to arginine to remove PEGylation sites that caused unacceptable reduction in biological activity. Lysine depletion approaches have been used to promote N-terminal^44^ or to have a single lysine residue remaining to promote site-selected conjugation.^44^–^46^ Use of lysine depletion methods is challenging because lysines are usually abundant^47^ and are necessary for protein activity.^45^,^48^–^50^ Strategies continue to be developed for lysine specific conjugation.^51^

**Fig. 1:**
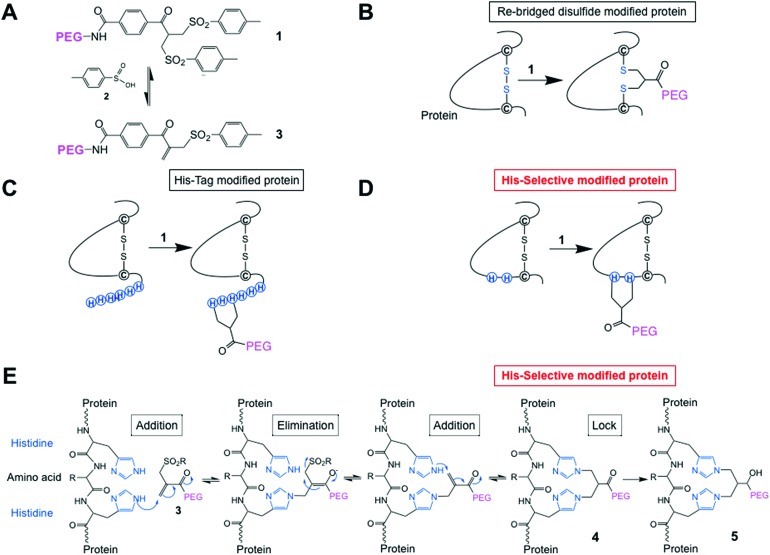
(A) Chemical structure of the PEG-bis-sulfone **1** and formation of the PEG-mono-sulfone reagent **3** generated after elimination of toluene sulfinic acid **2**; (B) both PEG-bis- and mono-sulfone reagents (**1** and **3**) can undergo site-specific bis-alkylation with the two cysteine thiols from a native disulfide in a protein achieving disulfide-bridging PEGylation;^52^ (C) both reagents (**1** and **3**) also undergo conjugation to the histidines in a C- or N-terminal histidine tag (His-tag);^64^ (D) herein we investigated site-selective bis-alkylation conjugation at a His_2_-tag engineered at a selected site along the protein mainchain; (E) mechanism for conjugation by bis-alkylation at a His–Gly–His tag by a sequence of addition–elimination reactions to the initial conjugate **4**. Carbonyl reduction to give **5** stops de-PEGylation by preventing retro-Michael reactions.

We have found that the PEG-bis-sulfones **1** can undergo site-specific bis-alkylation with the two thiols from a native disulfide bond in a protein (Fig. 1A and B).^52^,^53^ The bis-sulfone **1** undergoes elimination of a toluene sulfinic acid (*e.g.***2**) to the mono-sulfone **3** which is then capable of undergoing a sequence of addition–elimination reactions to site-specifically modify the protein by re-bridging a reduced disulfide with a three carbon bridge attached to PEG. Other bridging reagents have been described,^54^–^57^ and we have continued to examine reagents based on the PEG-bis-sulfone **1** due to the simplicity of their preparation and their practical application to prepare ADCs^58^–^60^ and antibody mimetics.^61^–^63^

We have also found that the PEG bis- and mono-sulfones (**1** and **3**) can site-specifically undergo conjugation to the histidines in a C- or N-terminal histidine tag (His-tag) (Fig. 1C)^64^ without need of metal chelation. His-tags are a contiguous sequence of histidines (*e.g.* six histidines, His_6_-tag), which are widely used in a broad range of applications in protein science and enable effective purification and can also increase protein expression yields.^65^,^66^ His-tags are usually placed at a C- or N-terminus of a protein where often one or both of these regions of a protein do not adversely compete with protein function, *e.g.* binding or activity. Our reasoning was that PEGylation at a terminal His-tag would display higher activity, which indeed is what was observed with interferon α2-a and a domain antibody.^64^ Another way to target His-tags for conjugation is to use reagents chelated to nickel or zinc which can then complex to the imidazoles in a His-tag on a protein. Applications tend to be for protein labeling and immobilisation.^67^–^69^ Covalent bonds can be formed after complexation by subsequent reaction by other functionality on the reagent.^70^,^71^

During our studies of His-tag PEGylation,^64^ preliminary experiments were also conducted with a small peptide called chemerin-9 peptide (149-YFPGQFAFS-157). Varying numbers of histidines (*e.g.* two, four or six) and a histidine–glycine tag (**H**G**H**G**H**G) were appended to the C-terminus of the peptide. Chemerin-9 peptide does not contain any lysines and PEGylation was only observed when histidines were added to the peptide. We now examine the site-selective conjugation of PEG-mono-sulfone **3** to a truncated histidine tag comprised of 2 histidines (His_2_-tag) engineered into a protein that also has several lysine residues. Compared to a 6 histidine tag (His_6_-tag), a His_2_-tag could be positioned at more locations within the protein for site-selective conjugation while ensuring protein function can be maintained.

Most therapeutic proteins do not have contiguous histidines in their sequence, although some proteins are known to have clusters of histidines at their surface.^72^ While histidine is a relatively rare amino acid in proteins^73^ using bis-alkylation to target two histidines that are close together rather than a strategy to target one histidine will avoid the problem of competitive conjugation to other histidines in the protein of interest. There are three natural histidines and 11 lysine residues in interferon α2, none of which were conjugated when there was a N-terminal His-tag on interferon α2.^64^

Histidines have a lower p*K*_a_ than other nucleophilic residues in a protein, *i.e.*, lysine and arginine, so at mildly acidic pH (5–6) they may not be protonated, so can be reactive. Conjugation by bis-alkylation using reagents such as **1** and **3** can be equilibrium controlled *via* an addition–elimination reaction that is reliant on the Michael reaction. Covalent conjugation occurs with 2 amino acids close in space (*e.g.* the two cysteine thiols from a disulfide^53^ or two histidines in a C- or N-terminal his-tag^64^). Conjugation to two lysine amino nucleophiles in slightly acidic conditions is not favoured for reagents **1** and **3** when there are cysteine thiols or histidine imidazoles present. Therefore, using 2 closely placed histidines in interferon α2 (Fig. 1D and E) may provide an alternative method for achieving site-selective conjugation compared to, for example, (i) adding an unpaired cysteine to a protein with existing disulfides and (ii) incorporating ncAAs, which have been described for interferon α2.^15^,^74^

The aim of the study described herein was to determine if site-selected bis-alkylation conjugation could be accomplished with a two histidine tag (His_2_-tag) placed within the primary sequence of interferon α2-a to give a PEGylated interferon with a higher biological activity than has been observed previously for PEGylated interferon α2.

## Results and discussion

### Preparation of truncated N-terminal His-tag IFN analogues

Interferons are a group of naturally occurring cytokines produced in vertebrates in response to a viral infection.^75^ Cytokines are a broad group of proteins that have clinical relevance,^76^,^77^ but quickly clear from circulation, so several cytokines that have been approved for clinical use have also been PEGylated.

Interferons are a key component of the innate immune response and are used clinically to treat a wide range of conditions including viral infections, malignancies and multiple sclerosis. There are three types of interferon (I, II and III), and type-I interferons that are used clinically include interferon α and β. There are at least 13 different human interferon α proteins, all which have 166 amino acids except for interferon α2 which has 165 amino acids due to a deletion at position 44. Like many cytokines, interferon is a helical bundle protein that has a cluster of five α-helices, four of which are arranged to form a left handed helix bundle motif (helices A, B, C and E).^78^–^80^ Between helix A and B is a loop of 30 residues.

Initial experiments were conducted to determine if less than eight histidine residues at the N-terminus of interferon α2-a could undergo conjugation with PEG_10_-mono-sulfone **3** in conditions where native interferon α2-a was not PEGylated. The reagent **3** was derived from a PEG with a molecular weight of 10 kDa, so is abbreviated PEG_10_-mono-sulfone **3**. Interferon α2-a will herein be abbreviated to ‘IFN’.

It was first necessary to determine if a truncated N-terminal His_2_-tag IFN could undergo conjugation in conditions where native IFN did not undergo conjugation. Selective conjugation was thought more probable to occur in slightly acidic conditions to avoid lysine conjugation, so reactions were conducted with the PEG_10_-mono-sulfone **3** rather than PEG_10_-bis-sulfone **1**. The use of the PEG mono-sulfone **3** undergoes conjugation directly without the need for elimination of the first molecule of toluene sulfinic acid **2** (Fig. 1A). The elimination of toluene sulfinic acid **2** is much slower at acidic pH values than at neutral and basic pH values.^53^

**Fig. 2:**
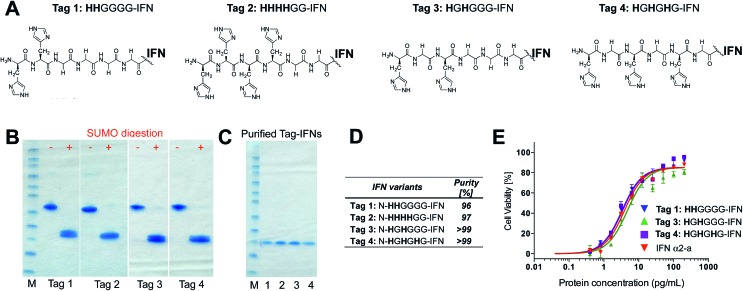
(A) Four N-terminal histidine tags were designed and fused to IFN to examine if different configurations of histidine residues would undergo selective conjugation with PEG_10_-mono-sulfone **3**; (B) SDS–PAGE gels (colloidal blue) of the N-terminally histidine tagged IFN–SUMO fusion proteins that were isolated and then digested to separate the SUMO protein from the IFN variant; (C) SDS–PAGE gel (colloidal blue) of the purified N-terminal IFN variants; (D) purities of the N-terminal IFN variants as determined by RP-HPLC. Only small amounts of the Tag 2 (**HHHH**GG-IFN) variant could be isolated, so was not used in the conjugation studies. (E) The three N-terminally tagged IFN variants (Tags 1, 3 and 4) used for conjugation studies displayed equivalent activity compared to native IFN.

Although it was not clear which IFN variants would be expressed, we sought to prepare four different histidine-truncated tags (ESI†). The first two IFN variants were fused with two or four contiguous histidines followed by glycines. Tag 1 (**HH**GGGG-IFN) and Tag 2 (**HHHH**GG-IFN) (Fig. 2A) were followed by glycines to make up a 6-amino acid tag analogous to a typical His_6_-tag commonly used in protein expression studies. The remaining two N-terminal IFN variants were prepared with a glycine separating the histidine residues: Tag 3 (**H**G**H**GGGG-IFN) and Tag 4 (**H**G**H**G**H**G-IFN) (Fig. 2A). Flexible protein inter-domains are rich in glycine residues.^81^ Our premise was that a His_2_-tag separated by a glycine would result in the flexibility needed to ensure the histidines would be positioned to achieve selective and efficient conjugation.

Initial expression experiments were not successful using the pET1010/D-TOPO® vector which had been used to prepare N-terminal 8-histidine tagged IFN (**H**_**8**_-IFN).^64^ We then examined IFN variants that were expressed as a fusion protein with the SUMO-tag using the Champion™ pET SUMO Expression system previously used to produce consensus interferon.^82^ The SUMO fusion of the four N-terminally tagged IFN variants were expressed on a 0.5 L scale using the SHuffle® *E. coli* expression strain.

The SUMO fusions of the N-terminally tagged IFN variants were expressed successfully in soluble form and were present in the supernatant fraction (ESI Fig. S1†). The SUMO fusion partner contains a His_6_-tag that allows affinity purification of the fusion protein prior to digestion needed to release the desired his-tagged IFN variant. Each SUMO–IFN variant was thus purified from the soluble fraction by immobilised metal affinity chromatography (IMAC) followed by digestion to remove the SUMO fusion partner (Fig. 2B). A second IMAC purification step was used as a negative purification step to separate the cleaved SUMO partner and yield the purified IFN variants (Fig. 2C). Purity of isolated IFN was confirmed by RP-HPLC (Fig. 2D, RP-HPLC are shown in the ESI†).

The Tag 2 variant (**HHHH**GG-IFN) with four contiguous histidine residues was biologically active (Table 1) but was difficult to purify at high enough scale from the SUMO protein, which contains six contiguous histidine residues. Since the aim of these experiments was to select a His_2_-tag if possible, it was decided that initial conjugation studies would be conducted using the other three His-tagged IFN variants: (i) Tag 1 (**HH**GGGG-IFN), (ii) Tag 3 (**H**G**H**GGGG-IFN) and (iii) Tag 4 (**H**G**H**G**H**G-IFN). These three IFN variants all displayed comparable activity to the interferon α-2a NIBSC standard in the A549/EMCV antiviral *in vitro* biological assay (Fig. 2E, Table 1).

**Table 1:** Antiviral activity of N-terminally tagged IFN variants confirmed that fusion of a histidine–glycine tag to the N-terminus does not impact on IFN activity

Sample	Specific activity calculated to NIBSC IFNα-2a [MIU per mg]				
	IFN	Tag 1	Tag 2	Tag 3	Tag 4
		**HH**GGGG-IFN	**HHHH**GG-IFN	**H**G**H**GGG-IFN	**H**G**H**G**H**G-IFN
*n*	*n* = 3	*n* = 5	*n* = 5	*n* = 5	*n* = 5
MEAN	254.2	228.5	216.7	242.4	218.4
SEM	34.5	23.2	18.5	31.4	51.4

### Conjugation to the truncated N-terminal His-tag IFN variants

PEG conjugation studies with the three His-tagged IFN variants (Tags 1, 3 and 4) were conducted using PEG_10_-mono-sulfone **3**. The 10 kDa molecular weight PEG is comparable to that used in the clinic for PEG-Intron® (12.5 kDa PEG) and many prior studies indicate that a 10 kDa PEG was sufficiently large enough^83^ to surpass the molecular weight threshold needed to sterically shield the IFN to reduce its biological activity.

**Fig. 3:**
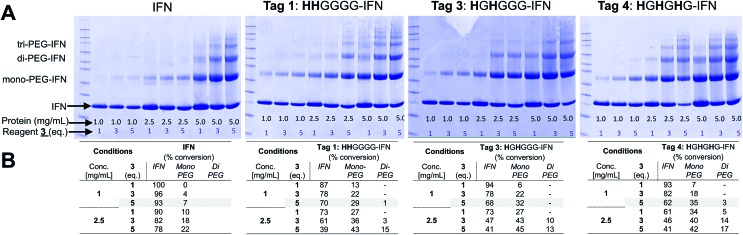
PEG conjugation studies of truncated N-terminal His-tagged IFN variants (Tags 1, 3 and 4) and native IFN used as control. Protein to PEG_10_-mono-sulfone **3** ratios were screened and the conjugation reaction was conducted for 16 h at 20 °C. It was observed that the most effective conjugation at the histidine-tag was achieved when reaction was conducted at pH 5.0 at a protein concentration of 1 mg mL^–1^ and 5 eq. of PEG_10_-mono-sulfone **3**. The conversion to the mono-PEGylated species ranged from 29–35% for the three IFN variants, while only 7% PEGylation on native IFN. (A) SDS–PAGE gels (colloidal blue) showing the PEG-conjugation reaction mixtures screened and (B) estimates of PEGylation conversion rates using densitometry analysis for the conjugation conducted with protein concentrations of 1.0 and 2.5 mg mL^–1^.

Native IFN^64^ was used as a control for these conjugation studies. When the conjugation reaction was conducted at pH 5.0 at a protein concentration of 1 mg mL^–1^ and 5 eq. of PEG_10_-mono-sulfone **3** for an incubation period of 16 h at 20 °C, the conversion to the mono-PEGylated species ranged from 29% to 35% for the three IFN variants (Tags 1, 3 and 4) whilst only 7% PEGylation was observed for the non-His-tagged IFN (Fig. 3A and B).

The **H**G**H**G**H**G-IFN variant (Tag 4) with three histidine residues did show an increased tendency to form di- and tri-PEGylated products when 5 equivalents of PEG_10_-mono-sulfone **3** was used in these conditions (*i.e.* incubated for 16 h at 20 °C). Multiple PEGylation is possible for the **H**G**H**G**H**G-IFN variant between the three histidines and the terminal amine. The **HH**GGGG and the **H**G**H**GGG-IFN variants with two histidine residues generated little or no di-PEGylated product. Increased protein concentration and PEG stoichiometry yielded unacceptable amounts of multi-PEG conjugates (Fig. 3A).

Previously with N-terminal octahistidine tagged IFN (**H**_**8**_-IFN)^64^ we found no evidence of PEG conjugation except on the His-tag suggesting histidine conjugation was faster at the optimal conditions than at other sites on the protein, and that once PEG was conjugated to the His-tag then it is possible that steric effects slowed the rate of non-specific conjugation.

Most optimised conjugation reactions require well defined and mild reaction conditions that are conducive to maintaining protein structure while achieving efficient and selective conjugation. At commercial scale, the cost of the GMP conjugation reagent is comparable or more expensive than the cost of the protein. Scalability considerations require that reagent stoichiometry, the amount of unmodified protein remaining after conjugation and the purification process used to isolate the desired conjugate should all be taken into account. Isolation of the desired conjugate from excess unmodified protein and conjugation reagent can generally be conducted effectively at scale.

Reductive amination strategies which require imine formation followed by mild reduction aim to exploit a small p*K*_a_ difference of the N-terminal amine for conjugation compared to the other amines in the protein. For PEG to be predominantly conjugated at the N-terminal amine,^84^ a narrow set of conditions that are protein dependent is required. Exploiting a slight difference in the p*K*_a_ of a single N-terminal amino acid to achieve high yielding and homogeneous imine formation in an aqueous medium is not possible with low reagent stoichiometry or complete site specificity.^85^,^86^ Several strategies have been described to direct conjugation to the N-terminus of a protein to address the inherent limitations of reductive amination using PEG aldehyde reagents.^13^,^64^,^87^

The data with the truncated N-terminal histidine tagged IFN variants (Fig. 3A and B) indicate that the best mono-PEGylation conditions were to incubate PEG_10_-mono-sulfone **3** (5 eq.) with the IFN variant at 1 mg mL^–1^ for 16 h at 20 °C (pH 5.0). Using these conditions, all three of the IFN histidine variants resulted in conjugation broadly comparable to that previously observed with an N-terminal **H**_**8**_-IFN.^64^ Purification of the N-terminal PEG–IFN conjugate was also easy to conduct. A single pair of histidines separated by a glycine, *e.g.***H**G**H**, was then incorporated within the IFN mainchain as a site-selective conjugation location.

### Site selection on IFN for histidine conjugation

Sites to incorporate an internal His-tag for conjugation were considered based on the published findings of IFN binding interactions with its dimeric receptor and the *in vitro* biological activities of isolated PEG-positional isomers.^88^,^89^ IFN exerts its biological activity through binding to the specific cell surface receptors.^90^ Features of the IFN structure and its interaction with cell membrane receptors have been described and key residues mediating these interactions have been determined.^91^ Type 1 IFNs bind to a heterodimeric cell surface receptor (IFNAR1 and IFNAR2).^91^–^95^ The amino acid sequences 29–35, 78–95 and 123–140 are thought to bind to IFNAR1 and IFNAR2.^91^,^94^.

The two main clinically used PEGylated interferon products (PEG-Intron® and Pegasys®) are produced with PEGylation reagents that undergo non-specific conjugation on interferon α2 proteins that differ by one amino acid. Pegasys® is produced from interferon α2-a (lysine at position 23) and PEG-Intron® is produced from interferon α2-b (arginine at position 23).

PEG-Intron® is a 12 kDa PEG conjugate of interferon α2-b derived from a linear PEGylation reagent functionalised with an *N*-hydroxy-succinimide (NHS) active ester that undergoes non-selective acylation with protein nucleophiles (*e.g.* terminal amine, lysine amines). There are at least 13 mono-PEGylated interferon α2-b positional isomers in PEG-Intron® with PEG conjugated at lysine, serine and histidine residues, and at the N terminus (Cys1).^88^,^96^ Most of the observed activity is from the positional isomer where PEG is conjugated to His34 (or H34).^97^ Reaction of an NHS ester with the histidine imidazole side chain results in the formation of a hydrolytically labile carbonyl imidazolide. The H34 positional isomer is the most hydrolytically labile PEG positional isomer,^97^ so the observed activity of 37% for H34 positional isomer is likely due to the presence of de-PEGylated, unmodified interferon α2-b. The *in vitro* antiviral activity of the positional isomer at the amine terminus is ∼13%, however the activities of the other positional isomers that have been described are much lower (<10%).

Pegasys® is made using a lysine-derived branched PEG reagent with two 20 kDa PEG molecules linked *via* urethanes to the two lysine amines. An NHS active ester at the lysine carboxylate functions as the protein conjugating moiety which undergoes non-selective acylation with protein nucleophiles. Hydrolysis of NHS functionalised PEG reagents is reported to be competitive at pH values where amine conjugation can occur (*e.g.* pH 7.5–8.0).^98^ While an excess of 5 equivalents has been claimed to give a 45% conversion to mono-PEGylated IFN α-2a,^99^ other studies indicate the need to use a greater excess of NHS PEGylation reagents (10 eq.).^100^ Pegasys® comprises at least nine bioactive positional isomers, with four major isomers PEGylated at the lysine residues at positions 31, 21, 131 and 134.^89^,^101^,^102^ The *in vitro* activities of the positional isomers range from ∼2% to 12%.

**Fig. 4:**
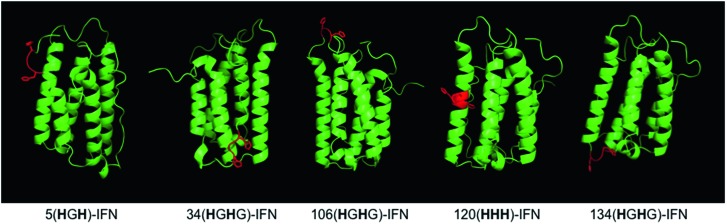
The IFN variants with an incorporated histidine conjugation tag (shown in red) were rationally designed based on the published findings on IFN binding interactions with its receptor and *in vivo* biological activities of the isolated PEG-positional isomers from marketed products.

Five different IFN variants with internal His-tags (or PEG-tags) were thus prepared (Fig. 4, Table 2, ESI†). The 5(**H**G**H**)-IFN variant was designed by engineering Q5H and T6G substitutions. One of the three natural histidine residues present within IFN is located at position 7, therefore this amino acid was not substituted. The resulting 5(**H**G**H**) conjugation tag is located within a short, flexible and solvent accessible loop in close proximity to the N-terminus of the protein. PEGylation of 5(**H**G**H**)-IFN allows comparison with the N-terminal **H**_**8**_-IFN conjugates previously described^64^ and the N-terminal PEG-Intron® positional isomer.

**Table 2:** Detailed description of the internal His-tagged IFN variants showing the localisation of His-tag insertion, sequence of the His-tag and native amino acids that were replaced

IFN	PEG-tag location	His-tag sequence	Original IFN amino acids
5(**H**G**H**)-IFN	5	**H**G**H**	QT**H**
34(**H**G**H**G)-IFN	34	**H**G**H**G	**H**DFG
106(**H**G**H**G)-IFN	106	**H**G**H**G	T-ET
120(**HHH**)-IFN	120	**HHH**	RKY
134(**H**G**H**G)-IFN	134	**H**G**H**G	K-YS

The 34(**H**G**H**)-IFN variant was prepared to evaluate if we could conjugate a PEG at the His34 (H34) site to compare with PEG-Intron®. The tag was incorporated through D35G and F36H substitutions. H34 is located on the AB loop and is thought to be involved in binding to the IFN receptor, thus it was anticipated that significant reduction of biological activity of native IFN would be observed.

The 106(**H**G**H**G)-IFN variant was prepared as this position is located within a flexible loop that is thought not to be involved with receptor binding. While glycosylation is rare among native human interferons, it is known that T106 can be O-glycosylated in interferon α2-b.^103^,^104^

Although IFN has two disulfides (C1–C98 and C29–C138), a study was conducted to insert a free cysteine at position 111 which is at the beginning of the D α-helix after the proline residue in this region.^15^ This cysteine IFN variant was PEGylated with maleimide PEG reagents and displayed comparable *in vitro* activity to PEG-Intron® in the Daudi cell growth inhibition assay. There are no reported PEG positional isomers at positions 99–109 in PEG-Intron® or PEGasys® due to the absence of nucleophilic amino acids in this region of the protein.^88^,^89^

The 106(**H**G**H**G)-IFN variant was created by making T106H, E107G and T108H substitutions. An additional G108 was inserted to allow more flexibility within the conjugation tag because there is a following proline in the IFN sequence. Proline restricts free rotation and typically appears on the surface of proteins while introducing a β-turn in the amino acid sequence.

Another IFN variant that was prepared is 120(**HHH**)-IFN, which was engineered with R120H, K121H and Y122H substitutions in the D α-helix. This tag is present in the D-helix which is solvent-exposed, but is less flexible compared to the loop regions where other IFN tags are located. The PEG positional isomer at lysine 121 (K121) in PEG-Intron® and PEGasys® displays <10% activity,^88^,^89^ so the 120(**HHH**)-IFN variant was prepared as a negative control. Three contiguous histidine residues (**HHH**) were used in this tag rather than a **H**G**H** tag to account for the lack of flexibility in the D-helix and to allow the imidazole rings to adopt a conformation for bis-alkylation conjugation.

The final tagged IFN variant, 134(**H**G**H**G)-IFN, was created using K134H, Y135G and S136H substitutions and insertion of additional glycine. The additional glycine was inserted because of the following proline residue in the sequence. The 134(**H**G**H**G) tag was created as another negative control to compare against the K134 positional isomer described during the development of Pegasys®,^89^ which had displayed low activity (<10%; 1358 ± 46 U μg^–1^).

Expression of the five internal His-tagged IFN variants was conducted using the SUMO system using SHuffle® *E. coli* strain (ESI†). No soluble expression was observed for 134(**H**G**H**)-IFN and this variant appeared to be present as insoluble protein aggregates at the top of SDS–PAGE well (Fig. S2†). This variant contains a tag in the binding region of IFN and was not expected to display increased *in vitro* antiviral activity once PEGylated over the other PEGylated IFN positional isomers described herein. No further attempts were made to express this protein.

**Fig. 5:**
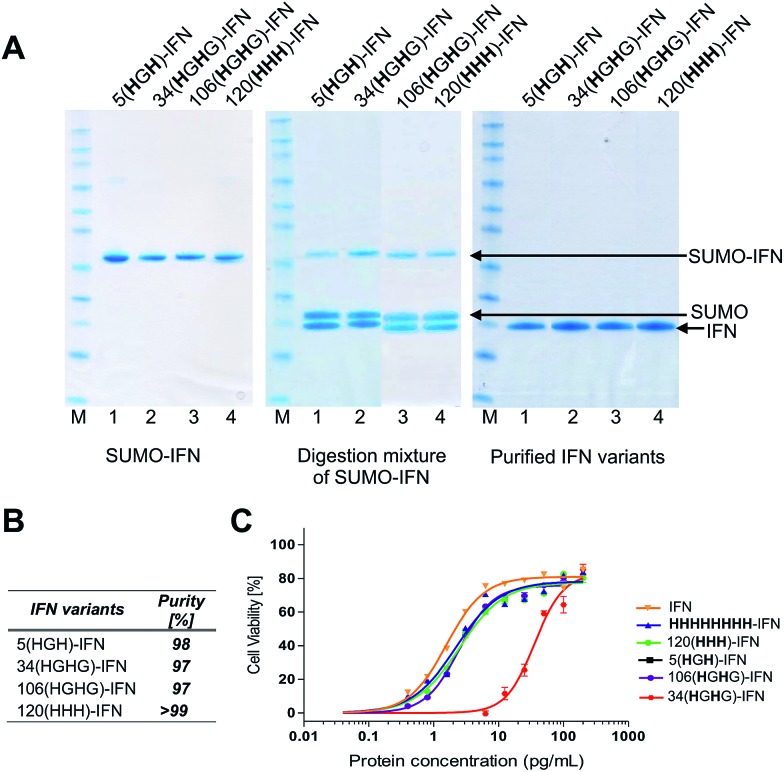
(A) SDS–PAGE gels (colloidal blue) of the five internal His-tagged IFN variants which had been expressed as IFN–SUMO-fusion proteins (colloidal blue detection). Proteolytic digestion with SUMO protease followed by IMAC purification yielded the designed IFN variants; (B) all of the histidine IFN variants were purified to high purity as determined by RP-HPLC; (C) antiviral activity showed that three histidine IFN variants had similar activity to native IFN, whereas the 34(**H**G**H**)-IFN variant was low (∼5%) compared to native IFN. The reduction in antiviral activity was not unexpected as the natural histidine located in position 34 in IFN is known to be important for IFN biological activity.

Fermentations (1 L) were conducted with the four remaining variants to give 90–110 mg of the SUMO–IFN protein per variant (ESI†). Each IFN variant was obtained after SUMO digestion and isolated by a sequential IMAC–anion exchange purification process. IMAC retained the SUMO fusion partner and the SUMO protease while allowing the elution of the IFN variants onto an anionic ion exchange chromatography (IEC) column. Each bound IFN variant was eluted at a concentration of ∼1 mg mL^–1^ to be used for conjugation studies (Fig. 5A) (ESI†). The four IFN variants were isolated in good purity (Fig. 5B, RP-HPLC are shown in the ESI†).

The process of protein engineering and verification of protein expression was shown to be straightforward and took less than two weeks. All of the expressed IFN variants remained soluble and stable following removal of the SUMO fusion partner. The expression method that was used to obtain the IFN variants did not require further optimisation from the method established for expression of native IFN.^82^ The *in vitro* antiviral activity of the four internal His-tagged IFN variants was determined using the A549/EMCV antiviral assay (Fig. 5C) using the NIBSC standard for IFN as control. The specific activities of the 5(**H**G**H**)-IFN, 106(**H**G**H**G)-IFN and 120(**HHH**)-IFN variants were similar to the NIBSC standard (Fig. 5C). The activity of the 34(**H**G**H**)-IFN variant was considerably lower (∼5% compared to native IFN) so this variant was not used in subsequent conjugation studies. The 34(**H**G**H**)-IFN variant was produced to compare with the H34 PEG positional isomer in PEG-Intron®.^97^ The reduced activity of 34(**H**G**H**)-IFN was not unexpected as the natural histidine located in position 34 in IFN is known to be important for biological activity.^96^,^105^–^107^

### Site-selective conjugation studies

**Fig. 6:**
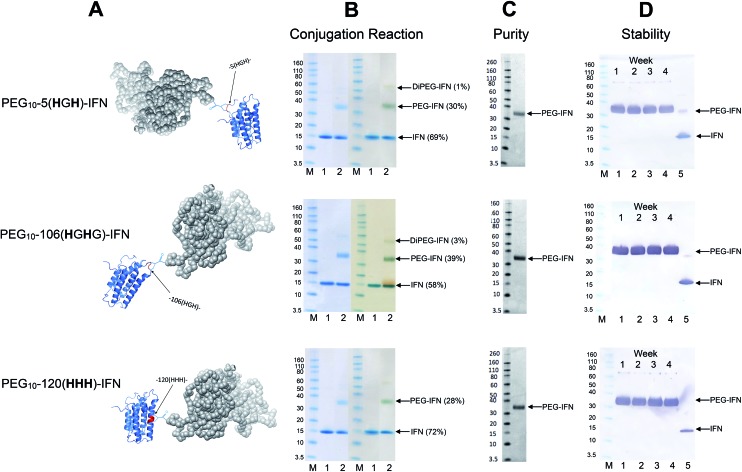
PEG conjugation studies were conducted with three of the internal His-tagged IFN variants (shown in A). PEG_10_-mono-sulfone **3** (5 eq.) was used for conjugation with each IFN variant at 1 mg mL^–1^. The conjugation reaction was conducted for 16 h at 20 °C and the observed conjugation conversions ranged between 28–39% and were similar to the N-terminally tagged IFNs. (B) SDS–PAGE gels for each IFN variant conjugation: lane M protein markers, lane 1, the IFN variant used for PEGylation and lane 2, the PEGylation reaction mixture. For each IFN variant, the left gel is stained with colloidal blue and the right gel is further stained with barium iodide. (C) Purified mono-PEGylated IFN variants were isolated from the crude conjugation reaction mixture using a single ion exchange purification step to deliver a final product in high purity as determined by SDS–PAGE (silver stain) and by RP-HPLC (Fig. S3†); (D) stability studies were conducted for 4 weeks at 4 °C in 50 mM sodium phosphate, 150 mM NaCl, pH 7.4 buffer with each PEG–IFN conjugate at 0.2 mg mL^–1^. All of the three site-selectively PEGylated IFN variants were stable to de-PEGylation with no evidence of free IFN being detected with anti-IFN western blot analysis, lane M protein markers, lane 1, week 1, lane 2, week 2, lane 3, week 3, lane 4, week 4 and lane 5, native IFN used as control.

Conjugation with PEG_10_-mono-sulfone **3** was examined with the three remaining IFN variants (Fig. 6A) using the conditions determined previously for the N-terminal truncated His-tagged IFNs (Fig. 3). The conjugation reactions were conducted with 5 molar equivalents of PEG_10_-mono-sulfone **3** and allowed to incubate overnight at 20 °C followed by analysis using SDS–PAGE (Fig. 6B). Conversions to the mono-PEGylated conjugates (28–39%) were similar to conversions observed during conjugation of the N-terminally His-tagged IFN variants (Fig. 3B).^64^ The stoichiometry of the PEG_10_-mono-sulfone **3** and the conversions to the PEG–IFN conjugate also compare favourably to other amine conjugation methods.^99^

Trace di-PEGylated product was sometimes observed by SDS–PAGE and was readily removed during purification. Non-specific PEGylation may have occurred in addition to PEGylation on the His-tag. Prior to purification, the PEGylated IFN variants were treated with sodium triacetoxyborohydride to reduce the aryl ketone to prevent retro-Michael induced PEG deconjugation.^64^,^108^ Carbonyl reduction using a mild hydride reagent acts as a stabilisation step and is comparable to the analogous process conducted during reductive amination^85^,^86^,^109^–^111^ in the manufacture of pegfilgrastim (Neulasta®).^84^ It has become evident even with maleimides that it is necessary to conduct a post-conjugation hydrolysis stabilisation step to avoid deconjugation.^112^–^114^

The mono-PEG conjugates were isolated from the crude reaction mixtures by a single IEC step. Fractions containing the majority of the 10 kDa mono-PEGylated IFN variants were combined (typically 7 mL) and then concentrated using a Vivaspin concentrator to 1 mL. Determination of the protein concentration for the three isolated PEG–IFN conjugates was accomplished using microBCA protein assay to calculate the final yields (Table 3).

**Table 3:** Final isolated yields for the site-selectively PEGylated IFN variants

Conjugate	Final yield [%]
**PEG**_10_-5(**H**G**H**)-IFN	25%
**PEG**_10_-106(**H**G**H**G)-IFN	33%
**PEG**_10_-120(**HHH**)-IFN	21%

Analysis by SDS–PAGE (silver stain detection) revealed there was no free IFN and RP-HPLC analysis indicated that the isolated conjugates were produced in high purity (Fig. S3† and 6C). No de-conjugation of PEG or free IFN was observed over a 28 day period for conjugates stored at 4 °C in 50 mM PBS, pH 7.4 (Fig. 6D, western blot analysis). Since SDS–PAGE analysis was consistent throughout,^32^ no MALDI analysis of the conjugates were conducted.

The fact that the mono-PEGylated IFN variant was by far the major PEGylated species produced during each conjugation reaction meant a simple one-step purification process based on cation exchange chromatography was sufficient to purify the desired PEG–IFN conjugate. The simple purification process gave essentially the pure mono-PEGylated IFN variant (>97% by RP-HPLC) in relatively high yields (based on conversion) ranging from 21–33%. This is an excellent recovery for the observed conjugation conversions (ranging from 28–39%).

**Fig. 7:**
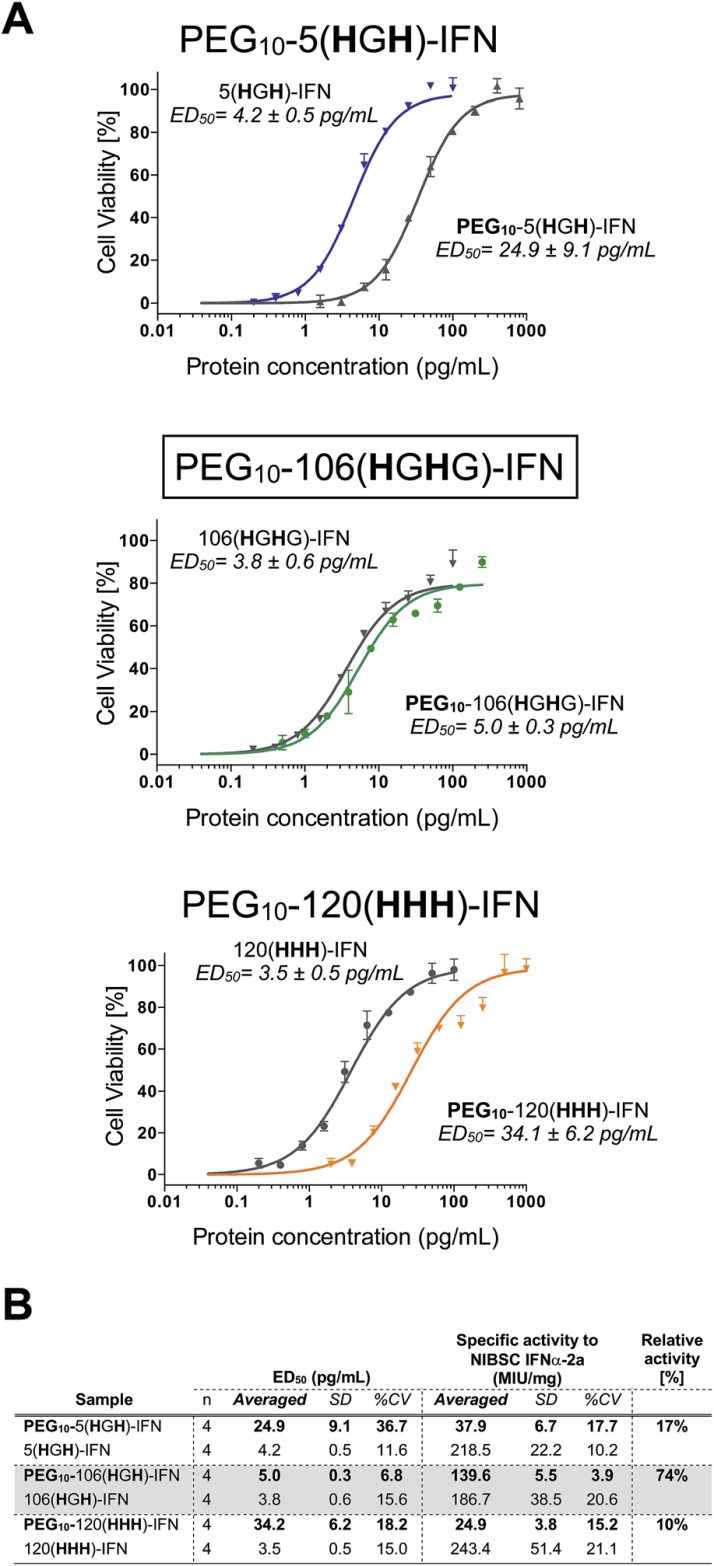
(A) Antiviral activity showed that all three of the internally histidine labelled PEG_10_-IFN variants were active, and that the level of retained activity significantly depends on the site of conjugation. (B) Antiviral activity of the site-specifically PEGylated IFN variants revealed that the site of PEG conjugation has significant effect on the level of biological activity retained.

All three PEG–IFN conjugates are biologically active (Fig. 7A and B) in the A549/EMCV antiviral assay with specific activities calculated against the NIBSC standard. The site of PEG conjugation influences biological activity. As IFN binds to a heterodimeric cell surface receptor, the conjugation site is important to minimise competitive steric shielding effects of the PEG with IFN binding to its receptor. The conjugates displayed activity in the following order: PEG_10_-106(**H**G**H**G)-IFN ≫ PEG_10_-5(**H**G**H**)-IFN > PEG_10_-120(**HHH**)-IFN.

The decreased activity observed with PEG_10_-120(**HHH**)-IFN (10%) is consistent with the activity observed for the PEG positional isomers in PEG-Intron® and Pegasys® when PEG was conjugated to the D α-helix at position 121. The activity was 9% for PEG-Intron® and 2.2% for Pegasys®.^88^,^89^ The greater reduction of bioactivity observed for Pegasys® may be due to the conjugation having been done with a branched PEG reagent (2 × 20 kDa PEG). The two separate PEG molecules, though hinged, appear to cause greater steric shielding of the receptor interaction site on IFN than a single linear PEG molecule.

The 17% activity of the PEG_10_-5(**H**G**H**)-IFN variant is similar to that of the N-terminal **H**_**8**_-IFN that had previously been PEGylated.^64^ Both PEG–IFN conjugates have the PEG near or at the N-terminus of the IFN, which further supports the importance of the conjugation site on biological activity. Similar activity of 10–16% was observed with a IFN variant with cysteine inserted at position 5 for conjugates derived from PEG reagents with molecular weights ranging from 10–40 kDa.^15^

The activity of the PEG_10_-106(**H**G**H**G)-IFN variant was similar to that of the un-PEGylated 106(**H**G**H**G)-IFN parent protein (5.0 ± 0.3 pg mL^–1^*vs.* 3.8 ± 0.6 pg mL^–1^). PEG_10_-106(**H**G**H**G)-IFN displayed an exceptionally high activity of 74% relative to the unPEGylated protein. The conjugation site in the 106(**H**G**H**G)-IFN variant is located away from known receptor binding sites in a flexible and solvent accessible loop, which makes it possible for retaining most of the bioactivity after PEGylation. This level of antiviral activity for a stable interferon PEG conjugate is unprecedented. The PEG_10_-106(**H**G**H**G)-IFN example demonstrates that a His_2_-tag can serve as a selective site for bis-alkylation conjugation.

## Conclusions

Interferon α2-a variants were engineered with histidine conjugation tags. In most cases two histidines were separated by a glycine (*e.g.***H**G**H**) and this internal His-tag was placed at both the N-terminus and at different locations within the protein. We have termed these internal His-tags ‘PEG-tags’. Bis-alkylation PEGylation was used to give conjugates that displayed *in vitro* antiviral activity that was dependent on the site of the PEG-tag. PEGylation was conducted with bis-alkylation reagent **3** derived from PEG with a molecular weight of 10 kDa.

Since histidine is a natural amino acid, it was possible to use routine methods of site-directed mutagenesis to make the IFN variants which were expressed in soluble form to give similar titres obtained for native IFN. PEGylation conversions ranged from 28–39% and a single step purification process gave essentially the pure PEG–IFN variant (>97% by RP-HPLC) in high recovery with isolated yields ranging from 21–33%.

The level of retained bioactivity was strongly dependent on the site of PEG conjugation. The highest biological activity of 74% was retained for the PEG_10_-106(**H**G**H**G)-IFN which is unprecedented for a PEGylated IFN. The His_2_-tag was placed at a location in IFN where it was expected there would be minimal interference of PEG during binding with the interferon receptors. The biological activity for the PEG_10_-5(**H**G**H**)-IFN variant was 17% which is comparable to other PEGylated IFN conjugates at or near the N-terminus that have been described in the literature. The lowest retained activity (10%) was for PEG_10_-120(**HHH**)-IFN which was prepared as a negative control as this IFN variant was thought to be involved in receptor binding.

The presence of two histidines in PEG-tags to generate a target for bis-alkylating PEGylation is a feasible approach for site-selective PEGylation. The use of a PEG-tag strategically placed in a protein can result in maximising the retention of the biological activity after protein modification.

## Experimental

### Preparation of PEG_10_-mono-sulfone **3**

The PEG mono-sulfone **3** derived from 10 kDa PEG was used in these studies. The PEG bis-sulfone **1** was first prepared^115^ and was then incubated in 50 mM sodium phosphate buffer, pH 7.4, 2 mM EDTA, 150 mM NaCl at concentration of 10 mg mL^–1^ for a period of 6 h at 37 °C to provide the PEG mono-sulfone **3**.

### Site-selective PEGylation of IFN

Conjugation reactions were conducted on 1 mg scale of each IFN variant. The IFN proteins: (i) 5(**H**G**H**)-IFN (1.5 mg mL^–1^; 2 mL in 20 mM Tris, pH 8.0), (ii) 106(**H**G**H**G)-IFN (1.7 mg mL^–1^; 2 mL in 20 mM Tris, pH 8.0) and (iii) 120(**HHH**)-IFN (1.6 mg mL^–1^; 2 mL in 20 mM Tris, pH 8.0) were buffer exchange to an acetate-based PEGylation buffer (50 mM sodium acetate, pH 5.0 supplemented with 35 μM hydroquinone). Buffer exchange was conducted using a PD-10 desalting column (load: 2.5 mL) and eluted in 3.5 mL. The protein concentration determined by absorbance measurement at 280 nm were around 0.9 mg mL^–1^ for all samples (0.9 mg mL^–1^ for 5(**H**G**H**)-IFN; 0.92 mg mL^–1^ for 106(**H**G**H**G)-IFN and 0.9 mg mL^–1^ for 120(**HHH**)-IFN: 0.9 mg mL^–1^). The PEG mono-sulfone **3** prepared at 10 mg mL^–1^ was then added to each reaction mixture using 5 molar equivalents: 226 μL for 5(**H**G**H**)-IFN 231 μL for 106(**H**G**H**G)-IFN; and 225 μL for 120(**HHH**)-IFN. The conjugation reaction was allowed to incubate for 16 h at 20 °C and then the reaction mixtures were treated with 25 mM sodium triacetoxyborohydride (19 mg) which was added to the reaction mixture as a solid. The reaction mixture was allowed to incubate for 45 min on ice. This sequence of adding sodium triacetoxyborohydride and 45 min incubation was repeated twice.

The reaction mixture was then buffer exchanged into 50 mM sodium acetate, pH 4.0, using a pre-equilibrated PD-10 desalting column as previously described^64^ by loading 2.5 mL and eluting with 3.5 mL. The PD-10 column was again equilibrated with fresh buffer prior to the remaining 1.1 mL of reaction mixture being loaded, followed by addition of 1.4 mL of 50 mM sodium acetate, pH 4.0. The sample was then eluted with 3.5 mL of 50 mM sodium acetate, pH 4.0. The total volume of 7.5 mL of buffer exchanged reaction mixture was collected and subjected to ion exchange purification.

Cation exchange purification was performed on a MacroTrap SP HP (5 mL) column operated on an ÄKTAprime™ system. The column was firstly equilibrated with 30 mL of 50 mM sodium acetate, pH 4.0 (buffer A), followed by load of the PEGylation reaction mixture (7.5 mL). The flow-through was collected and the column was washed with 15 mL of buffer A to remove any residual PEG reagent. Subsequently, the column was washed with an increasing concentration of NaCl by applying a gradient elution of 50 mM sodium acetate, pH 4.0, 1 M NaCl (buffer B) from 0% to 100% typically over 30 min at 1 mL min^–1^. Fractions containing the desired product of the mono-PEGylated IFN conjugate from each variant were combined and concentrated to 1.0 mL using a Vivaspin centrifugal concentrator (MWCO 10 000, centrifuged at 3000*g* at 4 °C).

## Conflicts of interest

JWC is an employee of Abzena. KP, EL and RT no longer are employees of Abzena. SB was a co-founder of PolyTherics, a subsidiary of Abzena, but is not affiliated with either company.

## Supplementary Material

Supplementary information

## References

[cit1] Wilding K., Smith A., Wilkerson J., Bush D., Knotts T., Bundy B. (2018). ACS Synth. Biol..

[cit2] Pandey B., Smith M., Price J. (2014). Biomacromolecules.

[cit3] Shen B. Q., Xu K., Liu L., Raab H., Bhakta S., Kenrick M., Parsons-Reponte K. L., Tien J., Yu S., Mai E., Li D., Tibbitts J., Baudys J., Saad O. M., Scales S. J., McDonald P. J., Hass P. E., Eigenbrot C., Nguyen T., Solis W. A., Fuji R. N., Flagella K. M., Patel D., Spencer S. D., Khawli L. A., Ebens A., Wong W. L., Vandlen R., Kaur S., Sliwkowski S. X., Scheller R. H., Polakis P., Junutula J. R. (2012). Nat. Biotechnol..

[cit4] Francis M., Carrico I. (2010). Curr. Opin. Chem. Biol..

[cit5] Pasut G., Veronese F. M. (2012). J. Controlled Release.

[cit6] Turecek P., Bossard M., Schoetens F., Ivens I. (2016). J. Pharm. Sci..

[cit7] Sato H. (2002). Adv. Drug Delivery Rev..

[cit8] Rabuka D. (2010). Curr. Opin. Chem. Biol..

[cit9] Gauthier M. A., Klok H. A. (2010). Biomacromolecules.

[cit10] Thom J., Anderson D., McGregor J., Cotton G. (2011). Bioconjugate Chem..

[cit11] Hu Q., Berti F., Adamo R. (2016). Chem. Soc. Rev..

[cit12] Milczek E. (2017). Chem. Rev..

[cit13] Wang Y., Wu C. (2018). Biomacromolecules.

[cit14] Rosendahl M. S., Doherty D. H., Smith S. J., Carlson S. J., Chlipala E. A., Cox G. N. (2005). Bioconjugate Chem..

[cit15] Bell S. J., Fam C. M., Chlipala E. A., Carlson S. J., Lee J. I., Rosendahl M. S., Doherty D. H., Cox G. N. (2008). Bioconjugate Chem..

[cit16] Ohri R., Bhakta S., O'Donohue A., Cruz-Chuh J., Tsai S., Cook R., Wei B., Ng C., Wong A., Bos A., Farahi F., Bhakta J., Pillow T., Raab H., Vandlen R., Polakis P., Liu Y., Erickson H., Junutula J., Kozak K. (2018). Bioconjugate Chem..

[cit17] Trivedi M., Laurence J., Siahaan T. (2009). Curr. Protein Pept. Sci..

[cit18] Zhang L., Chou C., Moo-Young M. (2011). Biotechnol. Adv..

[cit19] Junutula J., Bhakta S., Raab H., Ervin K. E., Eigenbrot C., Vandlen R., Scheller R., Lowman H. B. (2008). J. Immunol. Methods.

[cit20] England P. (2004). Biochemistry.

[cit21] Connor R., Tirrell D. (2007). J. Macromol. Sci., Polym. Rev..

[cit22] Sletten E., Bertozzi C. (2009). Angew. Chem., Int. Ed..

[cit23] Zhang W., Otting G., Jackson C. (2013). Curr. Opin. Struct. Biol..

[cit24] Boutureira O., Bernardes G. J. L. (2015). Chem. Rev..

[cit25] Young D., Schultz P. (2018). ACS Chem. Biol..

[cit26] Schinn S., Bradley W., Groesbeck A., Wu J., Broadbent A., Bundy B. (2017). Biotechnol. Bioeng..

[cit27] Kalstrup T., Blunck R. (2015). Sci. Rep..

[cit28] Cho H., Daniel T., Buechler Y., Litzinger D., Maio Z., Putnam A., Kraynov V., Sim B., Bussell S., Javahishvili T. (2011). Proc. Natl. Acad. Sci. U. S. A..

[cit29] Wang Q., Wang L. (2008). J. Am. Chem. Soc..

[cit30] Martin R., BJ S., Kwon Y., Kay J., Davis R., Thomas P., Majewska N., Chen C., Marcum R., Weiss M., Stoddart A., Amiram M., Charna A., Patel J., Isaacs F., Kelleher N., Hong S., Jewett M. (2018). Nat. Commun..

[cit31] Debelouchina G. T., Muir T. W. (2017). Q. Rev. Biophys..

[cit32] Nischan N., Hackenberger C. (2014). J. Org. Chem..

[cit33] Harris Z., Gitlin J. (1996). Am. J. Clin. Nutr..

[cit34] Smith M., Easton M., Everett P., Lewis G., Payne M., RiverosMoreno V., Allen G. (1996). Int. J. Pept. Protein Res..

[cit35] Pickens C., Johnson S., Pressnall M., Leon M., Berkland C. (2018). Bioconjugate Chem..

[cit36] Wang A., Nairn N., Marielli M. and Grabstein K., in Protein Engineering, ed. P. Kaumaya, InTech, Rijeka, Croatia, 2012.

[cit37] Hamblett K., Senter P., Chace D., Sun M., Jenox J., Cerveny C., Kissler K., Bernhardt S., Kopcha A., Zabinski R. F., Meyer D., Francisco J. A. (2004). Clin. Cancer Res..

[cit38] Beck A., Goetsch L., Dumontet C., Corvaïa N. (2017). Nat. Rev. Drug Discovery.

[cit39] Sletten E., Bertozzi C. (2008). Org. Lett..

[cit40] Kozma E., Nikic I., Varga B., Aramburu I., Kang J., Fackler O., Lemke E., Kele P. (2016). ChemBioChem.

[cit41] Satomaa T., Pynnonen H., Vilkman A., Kotiranta T., Pitkanen V., Heiskanen A., Herpers B., Price L., Helin J., Saarinen J. (2018). Antibodies.

[cit42] Lyon R., Bovee T., Doronina S. O., Burke P., Hunter J., Neff-LaFord H., Jonas M., Anderson M., Setter J., Senter P. (2015). Nat. Biotechnol..

[cit43] Kopchick J. J., Parkinson C., Stevens E. C., Trainer P. J. (2002). Endocr. Rev..

[cit44] Yamamoto Y., Tsutsumi Y., Yoshioka Y., Nishibata T., Kobayashi K., Okamoto T., Mukai Y., Shimizu T., Nakagawa S., Nagata S., Mayumi T. (2003). Nat. Biotechnol..

[cit45] Metzger F., Sajid W., Saenger S., Staudenmaier C., Poel C., Sobottka B., Schuler A., Sawitzky M., Poirier R., Tuerck D., Schick E., Schaubmar A., Hesse F., Amrein K., Loetscher H., Lynch G., Hoeflich A., DeMeyts P., Schoenfeld H. (2011). J. Biol. Chem..

[cit46] Morishige T., Yoshioka Y., Inakura H., Tanabe A., Yao X., Tsunoda S., Tsutsumi Y., Mukai Y., Okada N., Nakagawa S. (2010). Biochem. Biophys. Res. Commun..

[cit47] deGraaf A., Kooijman M., Hennink W., Mastrobattista E. (2009). Bioconjugate Chem..

[cit48] Wiman B., Wallen P. (1977). Thromb. Res..

[cit49] Gitlin G., Bayer E., Wilchek M. (1987). Biochem. J..

[cit50] Rozen F., Pelletier J., Trachsel H., Sonenberg N. (1989). Mol. Cell. Biol..

[cit51] Matos M., Oliveira B., Martínez-Sáez N., Guerreiro A., Cal P., Bertoldo J., Maneiro M., Perkins E., Howard J., Deery M., Chalker J., Corzana F., Jiménez-Osés G., Bernardes G. (2018). J. Am. Chem. Soc..

[cit52] Shaunak S., Godwin A., Choi J. W., Balan S., Pedone E., Vijayarangam D., Heidelberger S., Teo I., Zloh M., Brocchini S. (2006). Nat. Chem. Biol..

[cit53] Balan S., Choi J. W., Godwin A., Teo I., Laborde C. M., Heidelberger S., Zloh M., Shaunak S., Brocchini S. (2007). Bioconjugate Chem..

[cit54] Smith M. E. B., Schumacher F. F., Ryan C. P., Tedaldi L. M., Papaioannou D., Waksman G., Caddick S., Baker J. R. (2010). J. Am. Chem. Soc..

[cit55] Li Z., Huang R., Xu H., Chen J., Zhan Y., Zhou X., Chen H., Jiang B. (2017). Org. Lett..

[cit56] Kuan S., Wang T., Weil T. (2016). Chem.–Eur. J..

[cit57] Bahou C., Richards D., Maruani A., Love E., Javaid F., Caddick S., Baker J., Chudasama V. (2018). Org. Biomol. Chem..

[cit58] Badescu G., Bryant P., Bird M., Henseleit K., Swierkosz J., Parekh V., Tommasi R., Pawlisz E., Jurlewicz K., Farys M., Camper N., Sheng X., Fisher M., Grygorash R., Kyle A., Abhilash A., Frigerio M., Edwards J., Godwin A. (2014). Bioconjugate Chem..

[cit59] Bryant P., Pabst M., Badescu G. O., Bird M., McDowell W., Jamieson E., Swierkosz J., Jurlewicz K., Tommasi R., Henseleit K., Sheng X., Camper N., Manin A., Kozakowska K., Peciak K., Laurine E., Gurygorash R., Kyle A., Morris D., Parekh V., Abhilash A., Choi J., Edwards J., Frigerio M., Baker M., Godwin A. (2015). Mol. Pharmaceutics.

[cit60] Pabst M., McDowell W., Manin A., Kyle A., Camper N., DeJuan E., Parekh V., Rudge F., Makwana H., Kantner T., Parekh H., Michelet A., Sheng X., Popa G., Tucker C. E., Khayrzad F., Pollard D., Kozakowska K., Resende R., Jenkins A., Simoes F., Morris D., Williams P., Badescu G., Baker M., Bird M., Frigerio M., Godwin A. (2017). J. Controlled Release.

[cit61] Khalili H., Khaw P., Lever R., Godwin A., Brocchini S. (2013). Bioconjugate Chem..

[cit62] Khalili H., Khaw P., Brocchini S. (2016). Biomater. Sci..

[cit63] Khalili H., Lee R., Khaw P., Brocchini S., Dick A. D. (2016). Sci. Rep..

[cit64] Cong Y., Pawlisz E., Bryant P., Balan S., Laurine E., Tommasi R., Singh R., Dubey S., Peciak K., Bird M., Sivasankar A., Swierkosz J., Muroni M., Heidelberger S., Farys M., Khayrzad F., Edwards J., Badescu G., Hodgson I., Heise C., Somavarapu S., Liddell J., Powell K., Zloh M., Choi J.-w., Godwin A., Brocchini S. (2012). Bioconjugate Chem..

[cit65] Woestenenk E. A., Hammarström M., Van den Berg S., Härd T., Berglund H. (2004). J. Struct. Funct. Genomics.

[cit66] Li M., Huang D. (2007). Protein Expression Purif..

[cit67] Tinazli A., Tang J., Caliokas R., Picuric S., Lata S., Piehler J., Liedberg B., Tampé R. (2005). Chem.–Eur. J..

[cit68] Hauser C., Tsien R. (2007). Proc. Natl. Acad. Sci. U. S. A..

[cit69] Lai Y., Chang Y., Hu L., Chao A., Du Z., Tanner J., Cheye M., Qian C., Ng K., Li H., Sun H. (2015). Proc. Natl. Acad. Sci. U. S. A..

[cit70] Meredith G., Wu H., Allbritton N. L. (2004). Bioconjugate Chem..

[cit71] Uchinomiya S., Nonaka H., Fujishima S., Tsukiji S., Ojida A., Hamachi I. (2009). Chem. Commun..

[cit72] Bolanos-Garcia V., Davies O. (2006). Biochim. Biophys. Acta, Gen. Subj..

[cit73] Kozlowski L. (2017). Nucleic Acids Res..

[cit74] Zhang B., Xu H., Chen J., Zheng Y., Wu Y., Si L., Wu L., Zhang C., Xia G., Zhang L., Zhou D. (2015). Acta Biomater..

[cit75] Isaacs A., Lindenmann J. (1957). Proc. R. Soc. B.

[cit76] Vilcek J., Feldmann M. (2004). Trends Pharmacol. Sci..

[cit77] Tayal V., Kalra B. (2008). Eur. J. Pharmacol..

[cit78] Presnell S., Cohen F. (1989). Proc. Natl. Acad. Sci. U. S. A..

[cit79] Kamtekar S., Hecht M. (1995). FASEB J..

[cit80] Klaus W., Gsell B., Labhardt A., Wipf B., Seen H. (1997). J. Mol. Biol..

[cit81] Polyak S., Forsberg G., Forbes B., McNeil K., Aplin S., Wallace J. (1997). Protein Eng..

[cit82] Peciak K., Tommasi R., Choi J., Brocchini S., Laurine E. (2014). Protein Expression Purif..

[cit83] Khalili H., Godwin A., Choi J., Lever R., Brocchini S. (2012). Bioconjugate Chem..

[cit84] Kinstler O., Molineux G., Treuheit M., Ladd D., Gegg C. (2002). Adv. Drug Delivery Rev..

[cit85] Cindric M., Cepo T., Galic N., Bukvic-Krajacic M., Tomczyk N., Vissers J. P. C., Bindila L., Peter-Katalinic J. (2007). J. Pharm. Biomed. Anal..

[cit86] Wang J., Hu T., Liu Y., Zhang G., Ma G., Su Z. (2011). Anal. Biochem..

[cit87] Rosen C., Francis M. (2017). Nat. Chem. Biol..

[cit88] Wang Y., Youngster S., Bausch J., Zhang R., McNemar C., Wyss D. (2000). Biochemistry.

[cit89] Foser S., Schacher A., Weyer K., Brugger D., Dietel E., Marti S., Schreitmuller T. (2003). Protein Expression Purif..

[cit90] The interferons: characterization and application, ed. A. Meager, Wiley-VCH Verlag, Weinheim, 2006.

[cit91] Quadt-Akabayov S., Chill J., Levy Y., Kessler N., Anglister J. (2006). Protein Sci..

[cit92] Bazan J. (1990). Proc. Natl. Acad. Sci. U. S. A..

[cit93] Pestka S. (2000). Biopolymers.

[cit94] Chill J., Quadt S., Anglister J. (2004). Biochemistry.

[cit95] Krause C., Pestka S. (2005). Pharmacol. Ther..

[cit96] Wang Y. S., Youngster S., Grace M., Bausch J., Wyss D. F. (2002). Adv. Drug Delivery Rev..

[cit97] Grace M., Youngster S., Gitlin G., Sydor W., Xie L., Westreich L., Jacobs S., Brassard D., Bausch J., Bordens R. (2001). J. Interferon Cytokine Res..

[cit98] Roberts M. J., Bentley M. D., Harris J. M. (2002). Adv. Drug Delivery Rev..

[cit99] Bailon P., Palleroni A., Schaffer C. A., Spence C. L., Fung W., Porter J. E., Ehrlich G. K., Pan W., Xu Z., Modi M. W., Farid A., Berthold W. (2001). Bioconjugate Chem..

[cit100] Basu A., Yang K., Wang M., Liu S., Chintala R., Palm T., Zhao H., Peng P., Wu D., Zhang Z., Hua J., Hsieh M., Zhang Z., Petti G., Li X. Y., Janjua A., Mendez M., Liu J., Longley C., Zhang Z., Mehlig M., Borowski V., Viswanathan M., Filpula D. (2006). Bioconjugate Chem..

[cit101] Dhalluin C., Ross A., Leuthold L., Foser S., Gsell B., Muller F., Senn H. (2005). Bioconjugate Chem..

[cit102] Dhalluin C., Ross A., Huber W., Gerber P., Brugger D., Gsell B., Senn H. (2005). Bioconjugate Chem..

[cit103] Adolf G., Kalsner I., Ahorn H., Maurer-Fogy I., Cantell K. (1991). Biochem. J..

[cit104] Nyman T., Kalkkinen N., Tolo H., Helin J. (1998). Eur. J. Biochem..

[cit105] Akabaov S., Biron Z., Lamken P., Piehler J., Anglister J. (2010). Biochemistry.

[cit106] Nedelman I., Akabayov S., Schnur E., Biron Z., Levy R., Xu Y., Yang D., Anglister J. (2010). Biochemistry.

[cit107] Foster G. (2004). Aliment. Pharmacol. Ther..

[cit108] Badescu G., Bryant P., Swierkosz J., Khayrzad F., Pawlisz E., Farys M., Cong Y., Muroni M., Rumpf N., Brocchini S., Godwin A. (2014). Bioconjugate Chem..

[cit109] Lee H., Jang H., Ryu S., Park T. (2003). Pharm. Res..

[cit110] Tong J., Zhong K., Tian H., Gao J., Xu X., Yin X., Yao W. (2010). Int. J. Biol. Macromol..

[cit111] Puchkov I. A., Kononova N. V., Bobruskin A. I., Bairamashvili D. I., Martyanov V. A., Shuster A. M. (2012). Russ. J. Bioorg. Chem..

[cit112] Lyon R. P., Setter J. R., Bovee T. D., Doronina S. O., Hunter J. H., Anderson M. E., Balasubramanian C. L., Duniho S. M., Leiske C. I., Li F., Senter P. D. (2014). Nat. Biotechnol..

[cit113] Morais M., Nunes J., Karu K., Forte N., Benni I., Smith M., Caddick S., Chudasama V., Baker D. (2017). Org. Biomol. Chem..

[cit114] Kalia D., Pawar S., Thopate J. (2017). Angew. Chem., Int. Ed..

[cit115] Brocchini S., Balan S., Godwin A., Choi J. W., Zloh M., Shaunaik S. (2006). Nat. Protoc..

