# Supplementary material for: Site-selective protein conjugation at histidine
Source: Chem Sci. 2018 Oct 9;10(2):427–39. doi: 10.1039/c8sc03355b (PMC6354831; doi:10.1039/c8sc03355b)
Supplement: Supplementary file 1 [file SC-010-C8SC03355B-s001.pdf]

Electronic Supplementary Material (ESI) for Chemical Science.
This journal is © The Royal Society of Chemistry 2018

Site-selective protein conjugation at histidine

Karolina Peciak[1,2], Emmanuelle Laurine[2], Rita Tommasi[2], Ji-Won Choi[2] and Steve Brocchini[1]

[1]UCL School of Pharmacy, University College London, 29-39 Brunswick Square, London, WC1N 1AX, UK

[2]Abzena plc, Babraham Research Campus, Babraham, Cambridge CB22 3AT, UK

ESI Figures

Figure S1

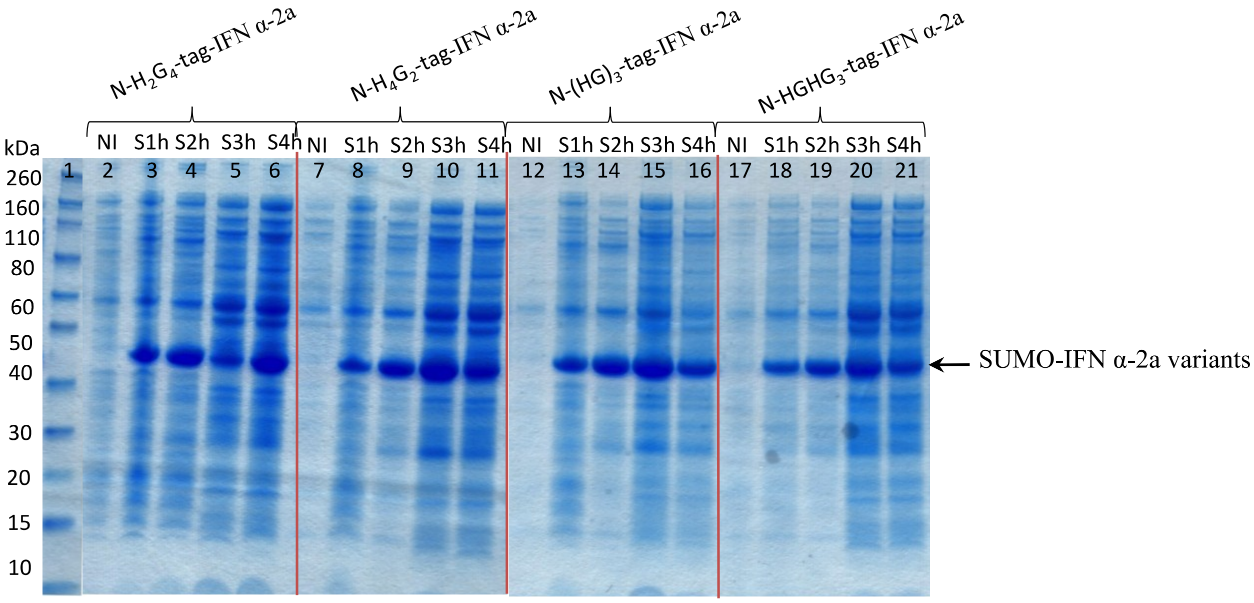

Protein stain

1. Novex sharp marker (3.5 µL)
2. N-H_2G_4-tag-IFN α-2a – non-induced (NI) - 6µL
3. N-H_2G_4-tag-IFN α-2a –Supernatant 1 h - 6µL
4. N-H_2G_4-tag-IFN α-2a – Supernatant 2 h - 6µL
5. N-H_2G_4-tag-IFN α-2a –Supernatant 3 h - 6µL
6. N-H_2G_4-tag-IFN α-2a – Supernatant 4 h - 6µL
7. N-H_4G_2-tag-IFN α-2a – non-induced (NI) - 6µL
8. N-H_4G_2-tag-IFN α-2a – supernatant 1 h - 6µL
9. N-H_4G_2-tag-IFN α-2a – supernatant 2 h - 6µL
10. N-H_4G_2-tag-IFN α-2a – supernatant 3 h - 6µL
11. N-H_4G_2-tag-IFN α-2a – supernatant 4 h - 6µL
12. N-$(HG)_3$-IFN α-2a – non-induced (NI) - 6µL
13. N-$(HG)_3$-IFN α-2a – supernatant 1 h - 6µL
14. N-$(HG)_3$-IFN α-2a – supernatant 2 h - 6µL
15. N-$(HG)_3$-IFN α-2a – supernatant 3 h - 6µL
16. N-$(HG)_3$-IFN α-2a – supernatant 4 h - 6µL
17. N-$HGHG_3$-IFN α-2a – non-induced (NI) - 6µL
18. N-$HGHG_3$-IFN α-2a – supernatant 1 h - 6µL
19. N-$HGHG_3$-IFN α-2a – supernatant 2 h - 6µL
20. N-$HGHG_3$-IFN α-2a – supernatant 3 h - 6µL
21. N-$HGHG_3$-IFN α-2a – supernatant 4 h - 6µL

Figure S1. Verification of expression of the SUMO fusion of the N-terminally tagged IFN variants: N-H_2G_4-IFN, N-H_4G_2-IFN, N-$(HG)_3$-IFN and N-$HGHG_3$-IFN using SHuffle ® T7 Express *E. coli* strain. Analysis of the supernatant (Sup) fractions withdrawn after 1h, 2h, 3h and 4h since induction of protein expression revealed that all of the SUMO fusions of the N-terminally tagged IFN variants were expressed successfully.

Figure S2

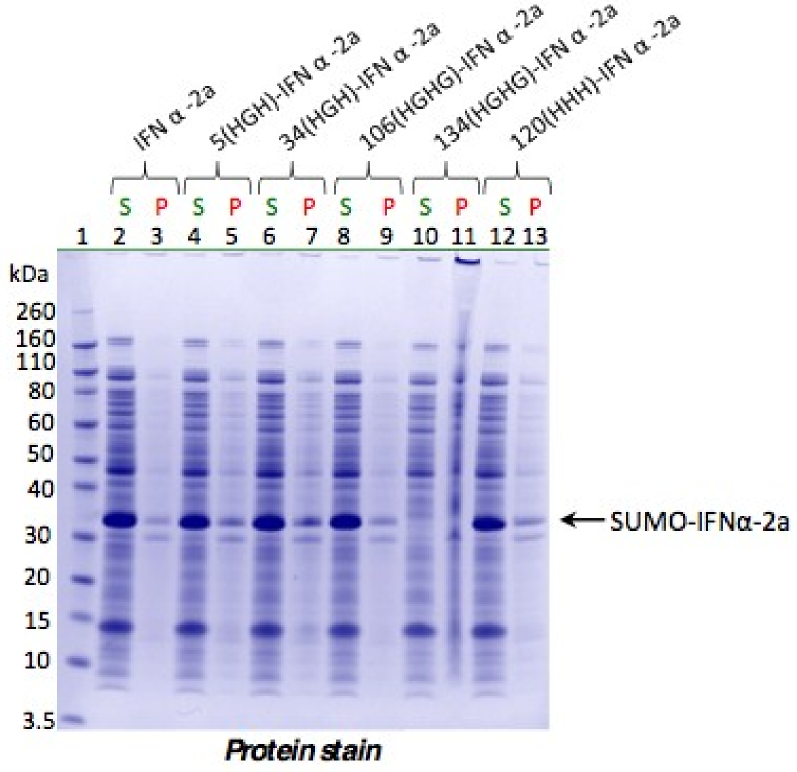

Figure S2. Recombinant SHuffle™ *E. coli* growth monitoring conducted in expression of the site-selectively histidine engineered IFN variants. Analysis of the soluble fraction (S) and insoluble pellet (P) at the collection time point revealed that except for 134(**H**G**H)**-IFN, the other four IFN variants were expressed in soluble form

Figure S3

(A)

(B)

(C)

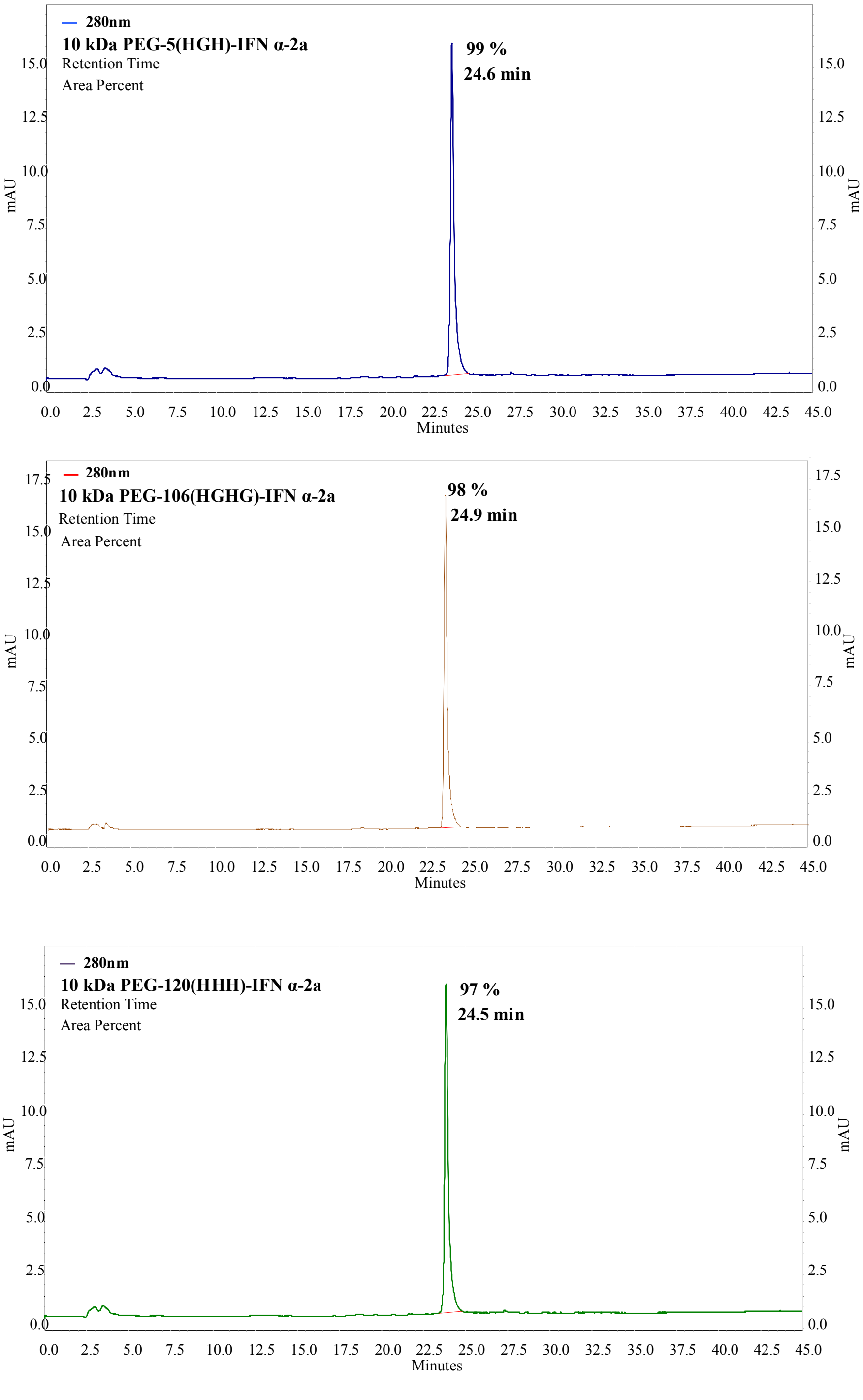

Figure S3. RP-HPLC analysis of the site-selectively PEGylated IFN variants: (A) PEG_{10}-5(**H**G**H**)-IFN, (B) PEG_{10}-106(**H**G**H**G)-IFN and (C) PEG_{10}-120(**HHH**)-IFN. The PEGylated IFN variants were isolated in good purity as determined by absorbance measurement at 280 nm.

Experimental

Design and preparation of the truncated N-terminal His-tagged IFN variants

The interferon α2-a (IFN) variants with four N-terminal truncated His$_2$-tags are listed below:

Tag 1: N-**HH**GGGG-IFN α-2a denoted as **H**$_2$G$_4$-IFN

Tag 2: N-**HHHH**GG- IFN α-2a denoted as **H**$_4$G$_2$ IFN

Tag 3: N-**H**G**H**GGG-IFN α-2a denoted as **H**G**H**G$_3$-IFN

Tag 4: N-**H**G**H**G**H**G-IFN α-2a denoted as (**H**G)$_3$-IFN

The IFN variants were based on the IFN α-2a sequence retrieved from the Protein Data Bank (accession number P01563) and optimised for expression in the *E. coli*. Native IFN α-2a (IFN) and N-terminal **HHHHHHHH**-IFN α-2a (**H$_8$**-IFN) were also prepared[1]. The full protein sequences of the IFN variants are shown below.

Tag 1: N-**HH**GGGG-IFN

```
        10         20         30         40         50         60
HHGGGGCDLP QTHSLGSRRT LMLLAQMRKI SLFSCLKDRH DFGFPQEEFG NQFQKAETIP

        70         80         90        100        110        120
VLHEMIQQIF NLFSTKDSSA AWDETLLDKF YTELYQQLND LEACVIQGVG VTETPLMKED

       130        140        150        160        170
SILAVRKYFQ RITLYLKEKK YSPCAWEVVR AEIMRSFSLS TNLQESLRSK E
```

Tag 2: N-**HHHH**GG-IFN

```
        10         20         30         40         50         60
HHHHGGCDLP QTHSLGSRRT LMLLAQMRKI SLFSCLKDRH DFGFPQEEFG NQFQKAETIP

        70         80         90        100        110        120
VLHEMIQQIF NLFSTKDSSA AWDETLLDKF YTELYQQLND LEACVIQGVG VTETPLMKED

       130        140        150        160        170
SILAVRKYFQ RITLYLKEKK YSPCAWEVVR AEIMRSFSLS TNLQESLRSK E
```

Tag 3: N-**HGH**GGG-IFN α-2a

```
        10         20         30         40         50         60
HGHGHGCDLP QTHSLGSRRT LMLLAQMRKI SLFSCLKDRH DFGFPQEEFG NQFQKAETIP

        70         80         90        100        110        120
VLHEMIQQIF NLFSTKDSSA AWDETLLDKF YTELYQQLND LEACVIQGVG VTETPLMKED

       130        140        150        160        170
SILAVRKYFQ RITLYLKEKK YSPCAWEVVR AEIMRSFSLS TNLQESLRSK E
```

Tag 4: N-**HGHGH**G-IFN

```
        10         20         30         40         50         60
HGHGGGCDLP QTHSLGSRRT LMLLAQMRKI SLFSCLKDRH DFGFPQEEFG NQFQKAETIP

        70         80         90        100        110        120
VLHEMIQQIF NLFSTKDSSA AWDETLLDKF YTELYQQLND LEACVIQGVG VTETPLMKED

       130        140        150        160        170
SILAVRKYFQ RITLYLKEKK YSPCAWEVVR AEIMRSFSLS TNLQESLRSK E
```

IFN

```
        10         20         30         40         50         60
CDLPQTHSLG SRRTLMLLAQ MRKISLFSCL KDRHDFGFPQ EEFGNQFQKA ETIPVLHEMI

        70         80         90        100        110        120
QQIFNLFSTK DSSAAWDETL LDKFYTELYQ QLNDLEACVI QGVGVTETPL MKEDSILAVR

       130        140        150        160
KYFQRITLYL KEKKYSPCAW EVVRAEIMRS FSLSTNLQES LRSKE
```

HHHHHHHH-IFN

```
        10         20         30         40         50         60
MHHHHHHHHC DLPQTHSLGS RRTLMLLAQM RKISLFSCLK DRHDFGFPQE EFGNQFQKAE

        70         80         90        100        110        120
TIPVLHEMIQ QIFNLFSTKD SSAAWDETLL DKFYTELYQQ LNDLEACVIQ GVGVTETPLM

       130        140        150        160        170
KEDSILAVRK YFQRITLYLK EKKYSPCAWE VVRAEIMRSF SLSTNLQESL RSKE
```

The pET SUMO expression system utilises a Small Ubiquitin-Like Modifier (SUMO), which has been demonstrated to enhance soluble protein expression. The pET SUMO expression vector was used previously to express soluble native IFN α-2a and IFN-con[2]. A set of oligonucleotide primers were designed to clone the N-terminal His-tagged IFN variants (**HH**GGGG-IFN**, HHHH**GG-IFN**, H**G**H**GGG-IFN and **H**G**H**G**H**G-IFN) into the pET SUMO

vector. The forward primer encoded for the chosen PEG-tag and one universal reverse primer was used for cloning all of the IFN variants (Table S1).

**Primer**	**Nucleotide sequence**	**Tm[°C]**
Forward:		
Tag 1: **HH**GGGG-IFN	**catcac**ggcggtggcggttgcgacc<br>**H H** G G G G C D	42°C
Tag 2: **HHHH**GG-IFN	**caccatcaccat**ggcggttgcgacc<br>**H H H H** G G C D	53°C
Tag 3: **H**G**H**GGG-IFN	**cac**ggc**cat**ggtggtggctgcgacc<br>**H** G **H** G G G C D	42°C
Tag 4: **H**G**H**G**H**G-IFN	**cac**ggc**cat**ggt**cac**ggctgcgacc<br>**H** G **H** G **H** G C D	48°C
Reverse:		
Reverse primer universal for all IFN α-2a	ttattactctttcgaacgcagagattcctgaagattgg	63°C

Table S1. List of primers designed for cloning of the N-terminal His-tagged IFN protein variants into the pET SUMO vector.

The PT-1 plasmid with a redesigned $\mathbf{H_8}$-IFN gene was used as a template to amplify the N-terminal His-tagged IFN variants. The PT1 plasmid was prepared for amplification by EcoRI restriction enzyme digestion conducted[2]. Gene amplification was conducted using Platinum® Taq DNA High Fidelity Polymerase to ensure production of the desired genes with a single 3 adenine overhangs required for ligation into the pET SUMO. The IFN gene amplification was initially conducted on a small scale (10 μL, five reactions) to screen a gradient of annealing temperatures (54.2°C to 70°C), followed by PCR amplification scale-up reaction (100 μL) amplified at 61°C and used for isolation of the desired genes. PCR amplification reactions were prepared as shown in (Table S2). The PCR program consisted of a DNA denaturation step at 95°C for 2 min followed by 30 cycles at 95°C for 40 s, 61°C for 90 s and 72°C for 1 min and a final elongation step at 72°C for 10 min.

Component	Quantity [μL]	
	Small scale	Scale-up
Plasmid template (1.5 ng/μL)	13.3 μL (20 ng)	26.6 μL (40 ng)
10× PCR Buffer	5 μL (1×)	10 μL (1×)
2.5 mM dNTPs	0.5 μL (0.25 mM)	1μL (0.25 mM)
Primer forward (30 μM)	1 μL (0.6 μM)	2 μL (0.6 μM)
Primer reverse (30 μM)	1 μL (0.6 μM)	2 μL (0.6 μM)
Platinum® Taq DNA Polymerase	0.2 μL (1 U)	0.4 μL (2 U)
Sterile water	29 μL	58 μL
Total	50 μL	100 μL

Table S2. Detailed components of PCR reaction used for optimisation of gene amplification and scale-up amplification of desired N-terminal His-tagged IFN protein variants.

The amplified N-terminal His-tagged IFN variants were purified using agarose gel electrophoresis followed by gene extraction and purification[2]. The isolated genes encoding for the N-terminal His-tagged IFN protein variants were then quantified using NanoDrop Spectrophotometer measurement at 260 nm (Table S3) and then used in ligation reaction with pET SUMO vector.

Gene	Concentration of isolated genes [ng/μL]
Tag 1: **HH**GGGG-IFN	34
Tag 2: **HHHH**GG-IFN	25
Tag 3: **H**G**H**GGG-IFN	32
Tag 4: **H**G**H**G**H**G-IFN	40

Table S3. List of amplified and isolated genes encoding for the N-terminal His-tagged IFN variants with concentrations of isolated genes.

The N-terminal His-tagged IFN genes were then cloned into the pET SUMO vector[2]. The PCR product (insert, I) and vector (vector, V) were mixed using a 1:1 molar ratio and the T4 DNA ligase was used for the ligation reaction as shown in Table S4. The resulting ligation reaction was incubated overnight at 20°C, followed by ligation reaction transformation into the TOP10 chemically competent *E. coli* cells.

Component	Quantity [μL]
PCR product (25 ng/mL)	3 μL (120 ng)
10× Ligation Buffer	1 μL
pET SUMO vector (25 ng/μL)	1 μL
Sterile water	-
T4 DNA Ligase	4 μL
Total	10 μL

Table S4. Ligation of the genes encoding for the **HH**GGGG-IFN, **HHHH**GG-IFN, **H**G**H**GGG-IFN and **H**G**H**G**H**G-IFN into the pET SUMO vector.

Recombinant colonies of each His-tagged IFN variant were selected based on kanamycin resistance. Thus, 100 μL of freshly transformed *E. coli* TOP10 cells with the pET SUMO ligation reaction of each IFN variant were plated onto an LB agar plate supplemented with kanamycin at a concentration of 50 μg/mL. The agar plates were then incubated overnight at 37°C. Several random colonies of each IFN construct were amplified in a 2 mL of LB medium supplemented with 50 μg/mL of kanamycin as previously described[2]. Subsequently, the DNA was extracted using the miniprep kit (Sigma Kit) as previously described[2] and eluted in 50 μL of sterile H_2O. The isolated plasmids were quantified using NanoDrop measurement at 260 nm (Table S5) and sent for DNA sequencing analysis performed at The Walston Institute, UCL.

Gene	Concentration of isolated pE SUMO-IFN α2a plasmids [ng/μL]
Tag 1: **HH**GGGG-IFN	120
Tag 2: **HHHH**GG-IFN	62
Tag 3: **H**G**H**GGG-IFN	114
Tag 4: **H**G**H**G**H**G-IFN	98

Table S5. Concentration of pET SUMO-IFN plasmids isolated from LB minipreps.

Verification of expression of the IFN variants cloned into the pET SUMO vector was conducted using SHuffle® T7 Express *E. coli* cell line. Plasmid selection was conducted based on kanamycin resistance (at 50 μg/mL), while protein expression was conducted for 4 h and was inducted with 1 mM IPTG. The bacterial pellet was next subjected to lysis and following conformation of all of the pET SUMO-IFN variants, the scale-up fermentation using the SUMO fusion system was as described for interferon consensus[2].

Preparation of the site-selectively engineered IFN variants

The internal His-tags, or PEG-tags, were selected based on information available in the literature about (i) IFN structure, (ii) IFN interactions with its cell membrane receptors and (iii) the *in vitro* biological activity of PEGylate IFN positional isomers. Five different locations within the IFN structure were selected for insertion of the histidine-based PEG-tag and the following IFN variants were produced (full sequences shown below and on the next page):

5(**H**G**H**)-IFN

34(**H**G**H**G)-IFN

106(**H**G**H**G)-IFN

134(**H**G**H**G)-IFN

120(**HHH**)-IFN

5(**HGH**)-IFN:

5(HGH)-IFNα vs. IFNα

98.8 % identity in 165 aa overlap

```
               10        20        30        40        50        60
5(HGH) CDLPHGHSLGSRRTLMLLAQMRKISLFSCLKDRHDFGFPQEEFGNQFQKAETIPVLHEMI
       ::::. ::::::::::::::::::::::::::::::::::::::::::::::::::::::
native CDLPQTHSLGSRRTLMLLAQMRKISLFSCLKDRHDFGFPQEEFGNQFQKAETIPVLHEMI
               10        20        30        40        50        60

               70        80        90       100       110       120
5(HGH) QQIFNLFSTKDSSAAWDETLLDKFYTELYQQLNDLEACVIQGVGVTETPLMKEDSILAVR
       ::::::::::::::::::::::::::::::::::::::::::::::::::::::::::::
native QQIFNLFSTKDSSAAWDETLLDKFYTELYQQLNDLEACVIQGVGVTETPLMKEDSILAVR
               70        80        90       100       110       120

              130       140       150       160
5(HGH) KYFQRITLYLKEKKYSPCAWEVVRAEIMRSFSLSTNLQESLRSKE
       :::::::::::::::::::::::::::::::::::::::::::::
native KYFQRITLYLKEKKYSPCAWEVVRAEIMRSFSLSTNLQESLRSKE
              130       140       150       160
```

34(**H**G**H**G)-IFN:

34(HGHG)-IFNα vs. IFNα

98.8 % identity in 165 aa overlap

```
               10        20        30        40        50        60
34HGHG CDLPQTHSLGSRRTLMLLAQMRKISLFSCLKDRHGHGFPQEEFGNQFQKAETIPVLHEMI
       ::::::::::::::::::::::::::::::::::  ::::::::::::::::::::::::
native CDLPQTHSLGSRRTLMLLAQMRKISLFSCLKDRHDFGFPQEEFGNQFQKAETIPVLHEMI
               10        20        30        40        50        60

               70        80        90       100       110       120
34HGHG QQIFNLFSTKDSSAAWDETLLDKFYTELYQQLNDLEACVIQGVGVTETPLMKEDSILAVR
       ::::::::::::::::::::::::::::::::::::::::::::::::::::::::::::
native QQIFNLFSTKDSSAAWDETLLDKFYTELYQQLNDLEACVIQGVGVTETPLMKEDSILAVR
               70        80        90       100       110       120

              130       140       150       160
34HGHG KYFQRITLYLKEKKYSPCAWEVVRAEIMRSFSLSTNLQESLRSKE
       :::::::::::::::::::::::::::::::::::::::::::::
native KYFQRITLYLKEKKYSPCAWEVVRAEIMRSFSLSTNLQESLRSKE
              130       140       150       160
```

106(**H**G**H**G)-IFN:

106(HGHG)-IFNα vs. IFNα
97.6 % identity in 166 aa overlap

```
               10        20        30        40        50        60
106HGH  CDLPQTHSLGSRRTLMLLAQMRKISLFSCLKDRHDFGFPQEEFGNQFQKAETIPVLHEMI
        ::::::::::::::::::::::::::::::::::::::::::::::::::::::::::::
native  CDLPQTHSLGSRRTLMLLAQMRKISLFSCLKDRHDFGFPQEEFGNQFQKAETIPVLHEMI
               10        20        30        40        50        60

               70        80        90       100       110       120
106HGH  QQIFNLFSTKDSSAAWDETLLDKFYTELYQQLNDLEACVIQGVGVHGHGPLMKEDSILAV
        :::::::::::::::::::::::::::::::::::::::::::::  .  :::::::::::
native  QQIFNLFSTKDSSAAWDETLLDKFYTELYQQLNDLEACVIQGVGVT-ETPLMKEDSILAV
               70        80        90       100        110

              130       140       150       160
106HGH  RKYFQRITLYLKEKKYSPCAWEVVRAEIMRSFSLSTNLQESLRSKE
        ::::::::::::::::::::::::::::::::::::::::::::::
native  RKYFQRITLYLKEKKYSPCAWEVVRAEIMRSFSLSTNLQESLRSKE
      120       130       140       150       160
```

120(**HHH**)-IFN:

120(HHH)-IFNα vs. IFNα
98.2% identity in 166 aa overlap

```
               10        20        30        40        50        60
120HHH  CDLPQTHSLGSRRTLMLLAQMRKISLFSCLKDRHDFGFPQEEFGNQFQKAETIPVLHEMI
        ::::::::::::::::::::::::::::::::::::::::::::::::::::::::::::
native  CDLPQTHSLGSRRTLMLLAQMRKISLFSCLKDRHDFGFPQEEFGNQFQKAETIPVLHEMI
               10        20        30        40        50        60

               70        80        90       100       110       120
120HHH  QQIFNLFSTKDSSAAWDETLLDKFYTELYQQLNDLEACVIQGVGVTETPLMKEDSILAVH
        :::::::::::::::::::::::::::::::::::::::::::::::::::::::::::.
native  QQIFNLFSTKDSSAAWDETLLDKFYTELYQQLNDLEACVIQGVGVTETPLMKEDSILAVR
               70        80        90       100       110       120

              130       140       150       160
120HHH  HHFQRITLYLKEKKYSPCAWEVVRAEIMRSFSLSTNLQESLRSKE
        ..:::::::::::::::::::::::::::::::::::::::::::
native  KYFQRITLYLKEKKYSPCAWEVVRAEIMRSFSLSTNLQESLRSKE
              130       140       150       160
```

134(**H**G**H**G)-IFN:

134(HGHG)-IFNα vs. IFNα
97.6 % identity in 166 aa overlap

```
               10        20        30        40        50        60
134HGH  CDLPQTHSLGSRRTLMLLAQMRKISLFSCLKDRHDFGFPQEEFGNQFQKAETIPVLHEMI
        ::::::::::::::::::::::::::::::::::::::::::::::::::::::::::::
native  CDLPQTHSLGSRRTLMLLAQMRKISLFSCLKDRHDFGFPQEEFGNQFQKAETIPVLHEMI
               10        20        30        40        50        60

               70        80        90       100       110       120
134HGH  QQIFNLFSTKDSSAAWDETLLDKFYTELYQQLNDLEACVIQGVGVTETPLMKEDSILAVR
        ::::::::::::::::::::::::::::::::::::::::::::::::::::::::::::
native  QQIFNLFSTKDSSAAWDETLLDKFYTELYQQLNDLEACVIQGVGVTETPLMKEDSILAVR
               70        80        90       100       110       120

              130       140       150       160
134HGH  KYFQRITLYLKEKHGHGPCAWEVVRAEIMRSFSLSTNLQESLRSKE
        :::::::::::::. ..:::::::::::::::::::::::::::::
native  KYFQRITLYLKEKK-YSPCAWEVVRAEIMRSFSLSTNLQESLRSKE
              130        140       150       160
```

The IFN variants were cloned using the QuikChange II XL Site-Directed Mutagenesis Kit (Agilent Technologies) to introduce the desired point mutations into the pET SUMO-IFN expression vector. This cloning system requires the template DNA plasmid to be isolated from dam+ *E. coli* strains, which ensures that the template plasmid is methylated. Following temperature cycling, the product is treated with Dpn I endonuclease specific for digestion of methylated and hemimethylated DNA. The Dpn I endonuclease is used to digest the template DNA plasmid, whilst the synthetised nicked DNA plasmid incorporating the desired mutations is transformed into *E. coli*, where nicked plasmid is repaired and amplified. The pET SUMO-IFN supercoiled double-stranded DNA (dsDNA) plasmid was isolated from SHuffle® *E. coli* strain as previously described for N-terminal **H$_8$**-IFN[1].

The mutagenic oligonucleotide primers were designed for incorporation of the desired mutations within the IFN sequence. Both mutagenic primers (forward and reverse) were designed to contain the desired mutation and anneal to the same sequence on opposite strands of the pET SUMO-IFN template plasmid. Primers were designed using Translate accessed at ExPasy and were purchased at Invitrogen (primers listed in Table S6).

IFN variant	Primer	Nucleotide sequence
5(**H**G**H**)-IFN	Forward	gcgacctgcct**catggccac**agtttggggagtcgc<br>D L P **H G H** S L G S R
	Reverse	GCGACTCCCCAAACTGTGGCCATGAGGCAGGTCGC
34(**H**G**H**G)-IFN	Forward	cagctgcttgaag**catggtcat**gactttggcttcccac<br>S C L **K H G H** D F G F P
	Reverse	GTGGGAAGCCAAAGTCATGACCATGCTTCAAGCAGCTG
106(**H**G**H**G)-IFN	Forward	gggcgtcggcgtt**catggccatggt**cctctcatgaagg<br>G V G V **H G H G** P L M K
	Reverse	CCTTCATGAGAGGACCATGGCCATGAACGCCGACGCCC
120(**HHH**)-IFN	Forward	gcattctggcggtg**catcatcac**ttccagcgtattacactg<br>I L A V **H H H** F Q R I T L
	Reverse	CAGTGTAATACGCTGGAAGTGATGATGCACCGCCAGAATGC
134(**H**G**H**G)-IFN	Forward	cccaagcacagggac**catggccat**gcttctctttcag<br>L K E K **H G H G** P C A W
	Reverse	CCCAAGCACAGGGACCATGGCCATGCTTCTCTTTCAG

Table S6. List of primers designed for cloning of the site-selectively modified IFN α-2a variants using the QuikChange II XL Site-Directed Mutagenesis Kit and the pET SUMO-IFN α-2a plasmid.

Mutagenesis of the pET SUMO-IFN plasmid was conducted to construct each the pET SUMO plasmid encoding for each of the IFN variants. Each of the primers was suspended in sterile water (100 ng/μL) and the mutagenesis reaction was conducted as indicated in Table

S7. Mutagenesis was then conducted using a PCR program consisting of a DNA denaturation step at 95°C for 1 min followed by 18 cycles at 95°C for 50 s, annealing temperature of 60°C for 50 s and 68°C for 10 min with a final elongation step at 68°C for 7 min. Each mutagenesis reaction of the respective IFN variants was then subjected to Dpn I digestion. To each amplification reaction (50 μL) was added the Dpn I restriction enzyme (10 U). The reaction mixture was gently mixed by pipetting the solution up and down several times. Subsequently, the reaction mixture was spin down for 1 min at 1,500 *g* and then incubated still at 37°C for 1 h to digest the template pET SUMO-IFN plasmid (unmutated plasmid). The resulting reaction mixture (3 μL of each reaction) was then used in transformation of the TOP10 chemically competent *E. coli* cells.

**Component**	**Volume [μL]**
10 × reaction buffer	5 μL
pET SUMO-IFN vector (30 ng/μL)	0.5 μL (15 ng)
Forward primer (100 ng/μL)	1.2 μL (120 ng)
Revers primer (100 ng/μL)	1.2 μL (120 ng)
100 mM dNTP mix	1 μL
Sterile water	40 μL
PfuUltra HF DNA polymerase (2.5 U/μL)	1 μl (2.5 U)
Total	50 μL

Table S7. Components of the mutagenesis reaction mixtures used to amplify the modified IFN variants. The pET SUMO-IFN DNA plasmid was used as a template. Forward and reverse primers of each IFN variants were used for mutagenesis.

Recombinant colonies were selected based on kanamycin resistance (50 μg/mL) performed as previously described[2]. For this reason, 100 μL of the TOP10 *E. coli* cells transformed with each of the IFN variants were plated onto an LB agar plate supplemented with kanamycin (50 μg/mL). The plates were incubated overnight at 37°C. Several random colonies which grew on the LB agar plate (50 μg/mL of kanamycin) were amplified in a 2 mL of LB (50 μg/mL of kanamycin)[2]. Subsequently, the DNA was extracted using the miniprep kit (Sigma Kit) accordingly as previously described[2] and eluted into sterile H_2O (50 μL). The isolated plasmid was then analysed by DNA sequencing performed at the Walston Institute, UCL.

Verification of expression of the site-selectively modified IFN variants cloned into the pET SUMO vector was conducted using SHuffle® T7 Express *E. coli* cell line. Expression of all site-selectively engineered IFN variants cloned into the pET SUMO vector was conducted

using conditions described for the preparation of IFN-con[2]. Fermentation was typically conducted on 0.5 L scale. First, an overnight pre-culture was set up in 0.1 L of LB medium supplemented with kanamycin (at 50 μg/mL). The pre-culture was grown in shake flask overnight at 37°C at 210 rpm. On the next day the Terrific Broth (0.5 L) supplemented with kanamycin (at 50 μg/mL) was inoculated with the overnight pre-culture using 1:10 dilution factor, followed by culture growth conducted at 37°C, 210 rpm. Protein expression was induced with 1 mM IPTG at the point when the culture OD_{600nm} reached 4-6. Protein expression was conducted for 4 h at 30°C. Subsequently, the bacterial pellet was harvested by collecting the fermentation into 50 mL tubes and centrifugation at 3500 *g* for 30 min, 4°C. The bacterial pellet was collected, weighted and stored at -20°C until protein purification was conducted. Typical yields of the wet pallet produced for each IFN variant are shown in Table S8.

IFN variant	Expression vector	Expression conditions	Wet pellet yield [g/L]
**HH**GGGG-IFN	pET SUMO	SHuffle®, Protein expression induced with 1 mM IPTG at $OD_{600\,nm}$ ~ 5 Protein expression conducted for 4 h at 30°C	~ 28
**HHHH**GG-IFN			~ 33
**H**G**H**G**H**G-IFN			~ 31
**H**G**H**GGG-IFN			~ 29
5(**HGH**)-IFN			~ 32
34(**H**G**H**G)-IFN			~ 30
106(**H**G**H**G)-IFN			~ 32
120(**HHH**)-IFN			~ 31
134(**H**G**H**G)-IFN			~ 20

Table S8. Yields obtained during fermentation of SUMO fusion of the N-terminally tagged IFN variants and the site-selectively modified IFN variants.

Purification of the SUMO-IFN variants

Typically 3 g of bacterial pellet was re-suspended in 25 mL of lysis buffer (1× PBS, lyzozyme (0.4 mg/mL), protease inhibitor cocktail (1:500 dilution), DNase (0.02 mg/mL)) and then vortexed until a homogeneous mixture was obtained. This mixture was incubated for 1 h at 4°C with gentle rocking. The solution was then sonicated (three times 1 min sonication at 50%, 0.5 cycles) and an equivalent volume of PBST (1× PBS, 1% Triton×-100, 25 mL) was added to the sample. The sample was then incubated for an additional 1 h at 4°C with gentle rocking. The sample was centrifuged at 3,500 *g* for 45 min at 4°C to pellet the cellular debris and separate the supernatant.

Collected fractions (crude extract, pellet and supernatant) were then analysed on the 4%-12% SDS-PAGE and the collected supernatant fraction was passed through both 0.45 μm and 0.2 μm filters followed by IMAC purification which was conducted on the supernatant

fraction (~50 mL of each SUMO-IFN variant) using a HisTrap™ FF column (5 mL, GE Healthcare) operated on an ÄKTApurifier™ system. The column was equilibrated with PBS pH 7.4 (5 column volume, 25 mL) and then the supernatant supplemented with 20 mM imidazole (~50 mL) was loaded onto the column at 2 mL/min. Subsequently, the column was washed with PBS pH 7.4 (buffer A) until the absorbance monitored at 280 nm stabilised at the baseline (~100 mL) and then a step was conducted at 50 mM imidazole, PBS pH 7.4 (5% of buffer B, buffer B: 1 M imidazole in PBS pH 7.4, 50 mL). The SUMO fusion of each IFN variant was eluted with a linear gradient from 50 mM imidazole to 250 mM imidazole in PBS pH 7.4 (5%-25% buffer B over 30 min at 1 mL/min).

Collected fractions were then analysed by SDS-PAGE and fractions containing the SUMO-IFN fusion protein were pooled and quantified by UV280nm (1 mg/mL = 0.611). The purified SUMO-IFN fusion proteins were then subjected to the SUMO digestion process which was accomplished using conditions optimised for digestion of SUMO fusion of IFN-con[2]. The combined fractions containing the SUMO fusions of each IFN variant was buffer exchanged into PBS using a PD-10 desalting column. The buffer exchange step was conducted by typically loading each IFN variant (2.5 mL) onto the pre-equilibrated PBS PD-10 desalting column. Each sample was then eluted with 3.5 mL of PBS and the concentration protein in the eluted fraction was quantified by UV280nm measurement (1 mg/mL = 0.611 Ab 280 units) (Table S9).

IFN variant	Starting sample conc. [mg/L]	Load volume [mL]	Elution volume [mL]	Final sample conc. [mg/mL]
**HH**GGGG-IFN	1.6	2.5	3.5	1.1
**H**G**H**G**H**G-IFN	1.2	2.5	3.5	0.8
**H**G**H**GGG-IFN	1.5	2.5	3.5	1.0
5(**H**G**H**)-IFN	1.6	2.5	3.5	1.1
34(**H**G**H**G)-IFN	1.3	2.5	3.5	0.9
106(**H**G**H**G)-IFN	1.6	2.5	3.5	1.1
120(**HHH**)-IFN	1.8	2.5	3.5	1.2

Table S9. Buffer exchange SUMO-IFN fusion proteins.

SUMO digestion was typically performed on 3 mg scale of the respective SUMO fusion proteins (Table S10) using 350 U of SUMO protease (1 U/μL). The digestion was conducted at room temperature for 4 h with gentle end-to-end rocking followed by SDS-PAGE and ImageQuant™ analysis to confirm the digestion progress. Purification of the released IFN variants was performed using IMAC purification step performed. IMAC purification was conducted using a HisTrap™ FF column (5 mL, GE Healthcare) and ÄKTApurifier™ system.

IFN variant	SUMO-IFN sample in PBS [mL]	SUMO protease [mL]	SUMO buffer 10 × [mL]	Sterile water [mL]	Total [mL]
**HH**GGGG-IFN (1.1 mg/mL)	2.7	0.35	0.7	0.7	7.0
**H**G**H**G**H**G-IFN (0.8 mg/mL)	3.5	0.32	0.7	0.65	6.5
**H**G**H**GGG-IFN (1.0 mg/mL)	3.0	0.35	0.7	0.7	7.0
5(**H**G**H**)-IFN (0.9 mg/mL)	3.3	0.35	0.7	0.7	7.0
34(**H**G**H**)-IFN (0.9 mg/mL)	3.3	0.35	0.7	0.7	7.0
106(**H**G**H**G)-IFN (1.1 mg/mL)	2.7	0.35	0.7	0.7	7.0
120(**HHH**)-IFN (1.2 mg/mL)	2.5	0.35	0.7	0.7	7.0

Table S10. SUMO digestion of the SUMO-IFN fusion proteins to release the His-tagged IFN variants.

Prior to the IMAC purification step, the digestion reaction mixture of the N-terminal His-tagged IFN variants was buffer exchanged to 1×PBS. The desalted sample was then supplemented with 10 mM imidazole prior to sample loading onto the HisTrap™ FF column. The sample was supplemented with low concentration of imidazole (10 mM) to prevent binding of the N-terminal His-tagged IFN variants containing two to four histidine residues onto the HisTrap™ column, whilst enabling the effective binding of the His_6-tagged SUMO fusion partner. The sample was then loaded onto the HisTrap™ FF column pre-equilibrated with 10 mM imidazole PBS pH 7.4 (30 mL). The flow-through containing the N-terminal His-tagged IFN variants was collected (12 mL), and the column was washed with 50% buffer B (1M imidazole in PBS, 30 mL) to clean the column from the SUMO fusion partner and SUMO protease both containing a His_6-tag. The flow through fraction (typically 12 mL) was then concentrated using a Vivaspin 15 with a 10,000 MWCO (to 2 mL). The concentrated fraction (2 mL) containing the N-terminal His-tagged IFN variants was then quantified by microBCA and subjected protein characterisation studies.

The microBCA assay was generally performed on protein samples in a concentration range of 2-40 μg/mL. Prior to the microBCA assay, sample concentration was estimated by measurement of absorbance at 280 nm using NanoDrop. If necessary the sample was diluted to the required range (50 μg/mL to 2 μg/mL) range in an appropriate buffer (typically in PBS). A standard curve for the microBCA assay was prepared using a BSA and was included in each microBCA assay. The standard curve was prepared starting from 200 μg/mL and nine

2-fold dilutions. In a 15 mL falcon tube the working solution of the microBCA reagent was prepared by mixing 25 parts of reagent A, 24 parts of reagent B with 1 part reagent C. Next, 150 µL of each standard and tested sample was transferred to separate wells in triplicate on a 96 - well microplate, then 150 µL of working reagent was added to each well as speedily as possible. The microplate was then covered and incubated at 37°C for 2 h. Following that the absorbance was measured at 570 nm on a plate reader and the concentration of the tested sample was determined.

Purification of the site-selectively modified IFN variants consisted of IMAC purification conducted on a HisTrap™ FF (1 mL) combined with a subsequent IEC conducted on a HiTrap™ QHP column (1 mL). Modification to the purification procedure was adopted because higher protein concentration (>1 mg/mL) was required in the subsequent PEGylation step. Thus, the combined process of two purification columns allowed separation of the site-selective IFN variants from the SUMO fusion partner and simultaneous concentration of the desired IFN variant during the IEC step.

The digestion reaction mixture was diluted to 50 mL using buffer A (20 mM Tris pH 8.0, 10 mM imidazole). The sample was then loaded onto a HisTrap™ FF (1 mL) column screwed on top of a HiTrap™ QHP column (1 mL). Both columns were previously equilibrated with buffer A. Following sample loading (50 mL), the column was washed with buffer A to remove residual detergent present in the SUMO digestion buffer. The column was then washed until absorbance monitored at 280 nm stabilised at the baseline (20 mL). Following that, the IMAC column was disconnected and further purification was continued only on the HiTrap™ QHP column. This allowed the SUMO fusion partner and SUMO protease to bind onto the HisTrap™ column, while the released IFN variants were bound onto the IEC column. Elution of the IFN variants from the HiTrap™ QHP column was conducted by applying a gradient elution conducted with buffer B (20 mM Tris, pH 8.0, 1 M NaCl). The target protein was eluted using a gradient elution from 0% to 30% of buffer B run over 30 min at 1 mL/min. The column was then washed with 100% of buffer B and fractions collected in this purification process were analysed by SDS-PAGE.

Characterisation of the His-tag IFN variants

Following protein preparation, the His-tagged IFN variants were characterised by (i) RP-HPLC, (ii) SDS-PAGE and (iii) A549/EMCV antiviral assay. The antiviral assay was conducted using the NIBSC control for IFN and protein activity was expressed in IU/mg.

RP-HPLC analysis of the histidine IFN variants. The produced histidine IFN variants were analysed using RP-HPLC (Figures S4-5). Samples were analysed on a C8 column (Discovery® C8, 5 µm, column dimensions: 15 cm × 4.6 mm) at a flow rate of 1 mL/min. The column was first equilibrated with buffer C (2% acetonitrile, H_2O, 0.1% TFA) and then 20 µg

(1 mg/mL, 20 μL) of the sample was injected. The column was washed for 5 min with buffer C and then eluted with a linear gradient for 30 min from 0% to 100% buffer D (100% acetonitrile, H_2O, 0.1% TFA).

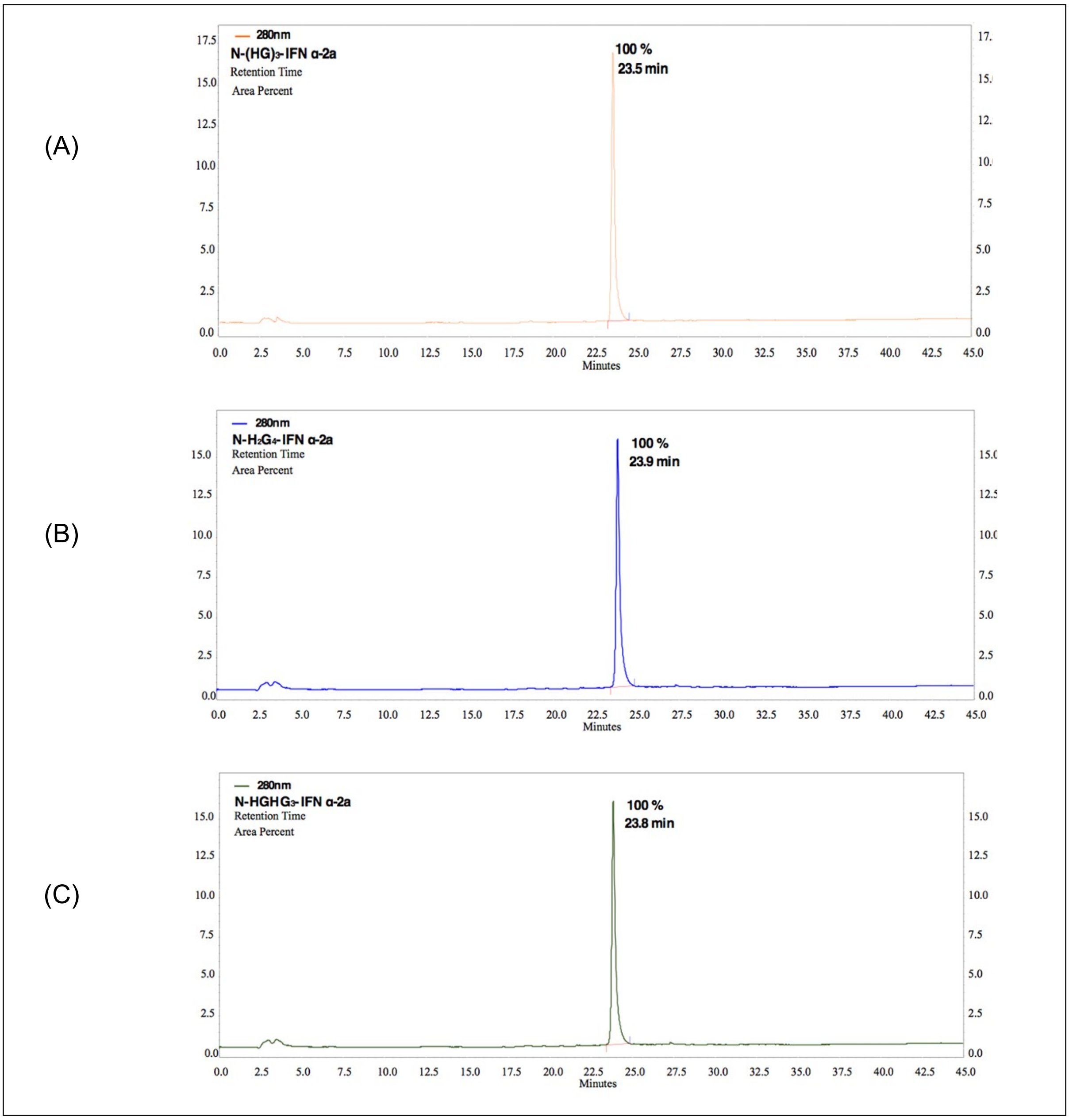

Figure S4. RP-HPLC analysis of the purified N-terminal His-tagged IFN variants: (A) **H**G**H**G**H**G-IFN (N-(HG)$_3$-IFN α-2a), (B) **HH**GGGG-IFN (N-H_2G_4)-IFN α-2a), (C) **H**G**H**GGG-IFN (N-HGHG3-IFN α-2a) indicated that all of the samples were isolated at good purity as determined by absorbance measurement at 280 nm.

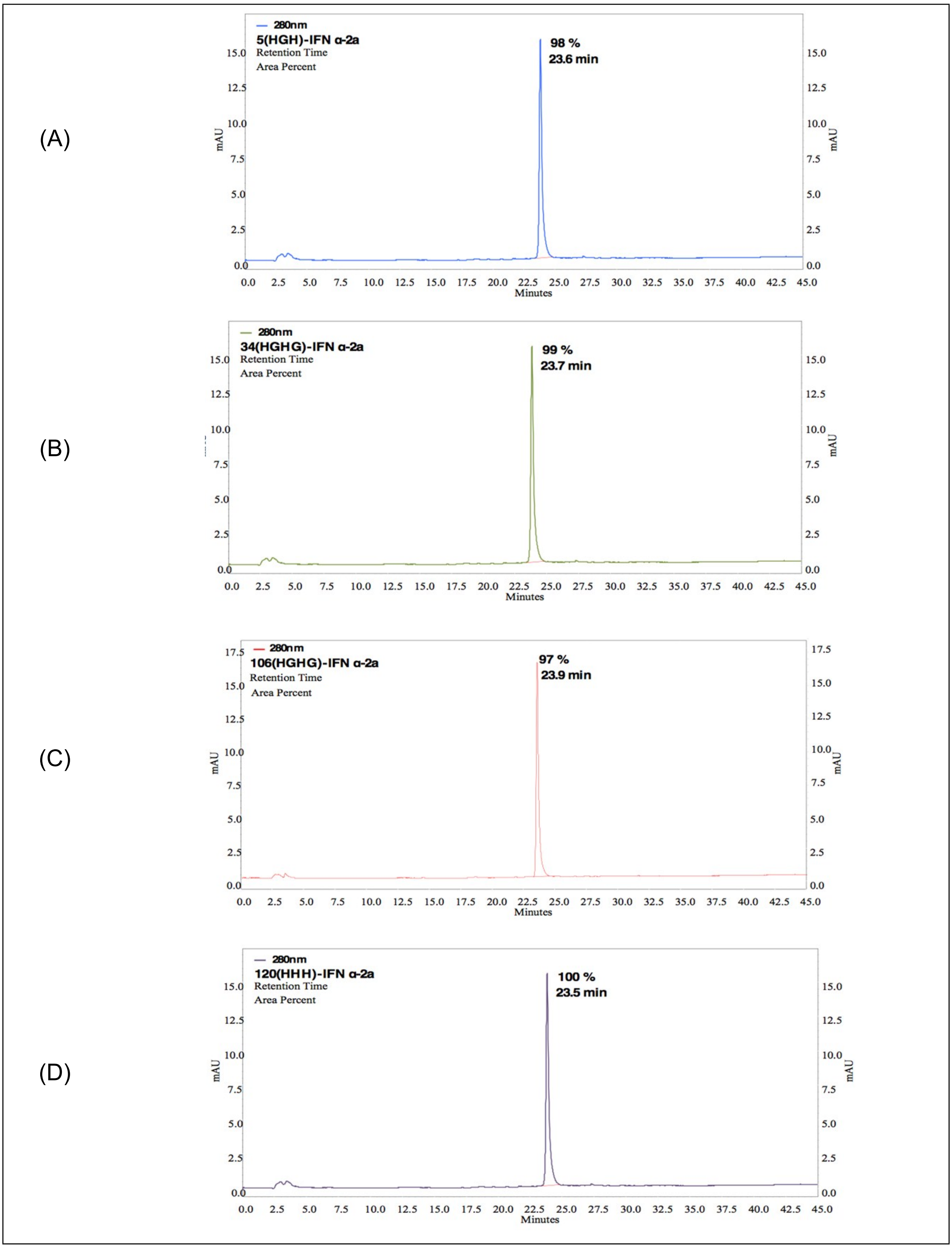

Figure S5. RP-HPLC analysis of the site-selectively engineered IFN variants: (A) 5(**H**G**H**)-IFN, (B) 34(**H**G**H**G)-IFN, (C) 106(**H**G**H**G)-IFN and (D) 120(**HHH**)-IFN indicated that all of the samples were isolated at good purity as determined by absorbance measurement at 280 nm.

Antiviral activity measurement. The biological activity of the produced His-tagged IFN variants (and their PEGylated products) was determined using an *in vitro* antiviral assay with human lung fibroblast cells (A549) and encephalomyocarditis virus (EMCV)[1, 3]. The A549 cell line was grown in the DMEM medium supplemented with 10% FBS, 50 units/mL of penicillin and 50 μg/mL of streptomycin. The A549 cells were firstly resuspended at the concentration of 17,000 cells/mL in the assay media (DMEM, 10% FBS, 50 units/mL of penicillin and 50 μg/mL streptomycin) and 50 μL/well of the cell solution was seeded in a 96-well flat-bottom microtiter plate (Nunc) plates. The plates were incubated overnight at 37°C with 5% CO_2.

On the next day a 2-fold dilution of the tested samples were added. The starting concentration of the test samples was determined experimentally to achieve a full cell viability curve with plateau reached at minimum and maximum concentrations tested and in the final optimised antiviral assays starting sample concentrations were 100 pg/mL for IFN. The potency was expressed as the concentration of the test substance that achieves 50% effective dose (ED_{50}), which in our assay corresponded to the protection against viral infection. It has been shown that the drawback of using ED_{50} values is that, due to different experimental conditions, ED_{50} values can differ across different laboratories[4]. Thus, specific activity values have been adopted internationally to overcome the need to standardise the potencies across different laboratories through the use of an internationally defined standards, such as NIBSC (National Institute for Biological Standards and Control). In our assay the NIBSC standards with known specific activity (code: 95/650) was used to normalise the ED_{50} values and to calculate the specific activities of the samples tested.

The sample was prepared in the assay medium (DMEM, 10% FBS, 50 units/mL of penicillin and 50 μg/mL streptomycin) and 50 μL/well of each dilution was added. The first and the last column in the plate were reserved for a positive and a negative control and at this point 50 μL/well of assay medium was added to those columns. Plates were incubated at 37°C with 5% CO_2 for 24 h.

After 24 h the medium was removed from the plates and cells were challenged with EMCV. The solution of EMCV virus was prepared in DMEM, 2% FBS, 50 units/mL penicillin and 50 μg/mL streptomycin (DMEM/2% FBS). The amount of the virus was optimised for a 19 h infection (20 μL of the virus was added to 20 mL of medium, virus titre = 1.6×10^5 Pfu/mL). The virus suspension (50 μL/well) was added to wells treated with the sample and to the positive control in the first and last columns of wells. DMEM/2% FBS was added to the negative control in the first and last column of the plate (50 μL/well). Plates were incubated at 37°C at 5% CO_2 for around 19 h until total lysis of cells in the negative control was observed. Plates were harvested by removing the medium and washing twice with sterile PBS (200 μL/well). To each well was then added 4% formaldehyde/0.1% methyl violet solution (50

µL/well) and the plates were incubated at room temperature for 20 min. The plates were then washed three times with PBS (200 µL/well) to remove cellular debris and the reminding cells were solubilised in 50 µL/well of 10% SDS followed by agitation. The absorbance measurement was taken at 570 nm using Opsys MR™ 96-well microplate reader. The results were analysed using GraphPad Prism 5 software. The potency was expressed as the concentration of the test substance that achieves 50% effective dose (ED_{50}), which in this assay corresponded to the protection against viral infection.

Optimisation of N-terminal His-tag PEGylation - small scale studies

Initial PEGylation studies were performed on a small scale (5 µg of protein) and was performed on $\mathbf{H_8}$-IFN and native IFN variants. Influence of the conjugation reaction parameters were evaluated and the aim was to select reaction conditions leading to *bis*-alkylation at the His-tag while there was trace, non-specific conjugation to native IFN[1]. The following reaction parameters were investigated: (i) reaction pH (4.0-7.5), (ii) protein concentration (1.0, 2.5 and 5.0 mg/mL) and (iii) PEG reagent equivalents (1, 3 and 5).

Small aliquots of the IFN variants were buffer exchanged to 10 mM ammonium bicarbonate, pH 8.0, using PD-10 (load: 2.5 mL, elution: 3.5 mL). The elution fraction (3.5 mL for each IFN variant) was then quantitated using UV measurement (280 nm) and protein concentration was calculated (1 mg/mL = 0.913). Subsequently, multiple aliquots of 25 µg, 12.5 µg and 5 µg of protein were prepared in a 0.2 mL lo-binding microfuge tubes. The samples were then freeze-dried and stored at -20°C until used for conjugation.

Influence of pH on PEGylation. Eight aliquots of freeze-dried $\mathbf{H_8}$-IFN and IFN containing 5 µg of each protein were re-suspended in 5 µL of the PEGylation buffers in varying pH (to final protein concentration 1 mg/mL). For pH values ranging from 4.0 to 5.5 the protein sample was resuspended in 50 mM sodium acetate buffer adjusted to pH 4.0, 4.5, 5.0, 5.3 and 5.5 and supplemented with 35 µM of hydroquinone. For pH values ranging from 6.0 to 7.5, the protein sample was resuspended in 50 mM sodium phosphate buffer adjusted to pH 6.0, 6.5, 7.0 and 7.5 and supplemented with 35 µM of hydroquinone. Subsequently, 3 eq. of the PEG-*mono*-sulfone **3** was added to the protein aliquots (Table S11). The conjugation reaction was then incubated without agitation at 20°C for 16 h, followed by SDS-PAGE and ImageQuant™ analysis.

Variant	Protein concentration (mg/mL)	Reagent <u>3</u> stoichiometry (eq.)	IFN MWt (Da)	Protein amount (μg)	Buffer volume (μL)	Reagent <u>3</u> concentration (mg/mL)	Reagent <u>3</u> volume (μL)
H_8-IFN	1	3	20497	5	5	10	0.73
IFN	1	3	19241	5	5	10	0.78

Table S11. Preparation of a small-scale PEGylation studies investigating influence of reaction pH on site-specificity of *bis*-alkylation.

Influence of protein concentration on PEGylation. Aliquots of freeze dried **H_8**-IFN and native IFN containing 25 μg, 12.5 μg or 5 μg of each protein were re-suspended in 5 μL of PEGylation buffer (50 mM sodium acetate, pH 5.0, 35 mM hydroquinine). Subsequently, varying equivalents of the PEG-*mono*-sulfone **<u>3</u>** was added to the protein aliquots at either 1, 3 or 5 equivalents (Tables S12-13). The conjugation reaction was then performed without agitation at 20°C for 16 h followed by SDS-PAGE and ImageQuant™ analysis.

Variant	Protein concentration (mg/mL)	Reagent <u>3</u> stoichiometry (eq.)	IFN MWt (Da)	Protein amount (μg)	Buffer volume (μL)	Reagent <u>3</u> concentration (mg/mL)	Reagent <u>3</u> volume (μL)
H_8-IFN	5	1	20497	25	5	10	1.2
H_8-IFN	5	3	20497	25	5	10	3.6
H_8-IFN	5	5	20497	25	5	10	6.1
H_8-IFN	2.5	1	20497	12.5	5	10	0.61
H_8-IFN	2.5	3	20497	12.5	5	10	1.8
H_8-IFN	2.5	5	20497	12.5	5	10	3.0
H_8-IFN	1	1	20497	5	5	10	0.2
H_8-IFN	1	3	20497	5	5	10	0.7
H_8-IFN	1	5	20497	5	5	10	1.2

Table S12. Small-scale PEGylation reactions using **H_8**-IFN to examine the influence of protein concentration and the stoichiometry of the PEG-*mono*-sulfone **<u>3</u>**.

Influence of reaction time and temperature on PEGylation. Aliquots of freeze dried **H_8**-IFN and native IFN were resuspended in 5 μL of the PEGylation buffer. The PEG *mono*-sulfone **<u>3</u>** (3 eq) was added to the protein aliquots (Table S13). The conjugation reaction was incubated without agitation at either 4 or 20°C for 1, 3 or 16 h followed SDS-PAGE and ImageQuant™ analysis.

Variant	Protein concentration (mg/mL)	Reagent <u>3</u> stoichiometry (eq.)	IFN MWt (Da)	Protein amount (μg)	Buffer volume (μL)	Reagent <u>3</u> concentration (mg/mL)	Reagent <u>3</u> volume (μL)
IFN	5	1	19241	25	5	10	1.2
IFN	5	3	19241	25	5	10	3.9
IFN	5	5	19241	25	5	10	6.5
IFN	2.5	1	19241	12.5	5	10	0.65
IFN	2.5	3	19241	12.5	5	10	1.9
IFN	2.5	5	19241	12.5	5	10	3.2
IFN	1	1	19241	5	5	10	0.3
IFN	1	3	19241	5	5	10	0.8
IFN	1	5	19241	5	5	10	1.3

Table S13. Small-scale PEGylation reactions using IFN to examine the influence of protein concentration and the stoichiometry of the PEG *mono*-sulfone **<u>3</u>**.

PEGylation of N-terminal His-tagged IFN variants. Aliquots of freeze dried **HH**GGGG-IFN, **H**G**H**GGG-IFN and **H**G**H**G**H**G-IFN were resuspended in 5 μL of the PEGylation buffer. Varying equivalents (1, 3, or 5) of the PEG-*mono*-sulfone **<u>3</u>** reagent were added to the protein aliquots (Table S14). The conjugation reaction was then incubated without agitation at 20°C for 16 h followed SDS-PAGE and ImageQuant™ analysis.

IFN α2a Variant	Protein concentration (mg/mL)	Reaget <u>3</u> stoichiometry (eq.)	IFN MWt (Da)	Protein amount (μg)	Buffer volume (μL)	Reagent <u>3</u> concentration (mg/mL)	Reagent <u>3</u> volume (μL)
**HH**GGGG-IFN	1	1	19743	5	5	10	0.25
**HH**GGGG-IFN	1	3	19743	5	5	10	0.75
**HH**GGGG-IFN	1	5	19743	5	5	10	1.26
**H**G**H**G**H**G-IFN	1	1	19903	5	5	10	0.25
**H**G**H**G**H**G-IFN	1	3	19903	5	5	10	0.75
**H**G**H**G**H**G-IFN	1	5	19903	5	5	10	1.26
**H**G**H**GGG-IFN	1	1	19823	5	5	10	0.25
**H**G**H**GGG-IFN	1	3	19823	5	5	10	0.75
**H**G**H**GGG-IFN	1	5	19823	5	5	10	1.26

Table S14. Preparation of a small-scale PEGylation studies conducted on the N-terminally his-tagged IFN variants: **HH**GGGG-IFN, **H**G**H**G**H**G-IFN and **H**G**H**GGG-IFN.

Characterisation of the PEG conjugates of the His-tagged IFN conjugates

The PEG conjugates of (i) 5(**H**G**H**)-IFN, (ii) 106(**H**G**H**G)-IFN and (iii) 120(**HHH**)-IFN were characterised to assess (i) sample purity by SDS-PAGE and RP-HPLC analysis, (ii) sample stability at 4°C for 4 weeks and (iii) bioactivity by A549/EMCV-based antiviral assay. Activity studies were conducted in parallel with the NIBSC standard for IFN. The antiviral activity of

the PEGylated conjugates was then expressed as specific activity calculated against the NIBSC standard. In addition, the percentage of the retained activity in respect to parental protein was determined.

Stability studies were performed in 50 mM sodium phosphate, 150 mM NaCl, pH 7.4 buffer with PEG-IFN conjugates at 0.2 mg/mL. The stability studies were performed for 4 weeks at 4°C during which samples were investigated weekly. All of the samples were supplemented with 1 mM sodium azide to inhibit bacterial growth and 0.2 mg/mL protease inhibitor to avoid the risk of protein degradation by proteases. At each time point 10 μL of the sample was drawn and stored at -80°C until all time points were collected and then analysed by SDS-PAGE followed by protein stain conducted with colloidal blue, PEG stain and anti-IFN western blot [2].

References

1. Cong Y*, et al.* Site-Specific PEGylation at Histidine Tags. *Bioconjugate Chem* **23**, 248-263 (2012).

2. Peciak K, Tommasi R, Choi J, Brocchini S, Laurine E. Expression of soluble and active interferon consensus in SUMO fusion expression system in E. coli. *Protein Expression Purif* **99**, 18-26 (2014).

3. Sjogren M*, et al.* Antiviral srsponse of HCV genotype 1 to consensus interferon and ribavirin versus PEGylated interferon and ribavirin. *Dig Dis Sci* **52**, 1540-1547 (2007).

4. Giard D, Fleischaker R. A study showing a high degree of interlaboratory variation in the assay of human interferon. *J Biol Stand* **12**, 265-269 (1984).
